# Biological Implications of Differential Expression of Mitochondrial-Shaping Proteins in Parkinson’s Disease

**DOI:** 10.3390/antiox7010001

**Published:** 2017-12-21

**Authors:** Sara Rocha, Ana Freitas, Sofia C. Guimaraes, Rui Vitorino, Miguel Aroso, Maria Gomez-Lazaro

**Affiliations:** 1 i3S—Instituto de Investigação e Inovação em Saúde, Universidade do Porto, 4200-135 Porto, Portugal; sara.rocha@i3s.up.pt (S.R.); anafreitas@ineb.up.pt (A.F.); sofia.guimaraes@i3s.up.pt (S.C.G.); miguel.aroso@i3s.up.pt (M.A.); 2 IBMC—Instituto de Biologia Molecular e Celular, Universidade do Porto, 4200-135 Porto, Portugal; 3 INEB—Instituto de Engenharia Biomédica, Universidade do Porto, 4200-135 Porto, Portugal; 4 FMUP—Faculdade de Medicina da Universidade do Porto, 4200-319 Porto, Portugal; 5 iBiMED, Department of Medical Sciences, University of Aveiro, 3810-193 Aveiro, Portugal; rvitorino@ua.pt; 6 Unidade de Investigação Cardiovascular, Departamento de Cirurgia e Fisiologia, Universidade do Porto, 4200-319 Porto, Portugal

**Keywords:** Parkinson’s disease, proteomics, bioinformatics, biological processes, mitochondria

## Abstract

It has long been accepted that mitochondrial function and morphology is affected in Parkinson’s disease, and that mitochondrial function can be directly related to its morphology. So far, mitochondrial morphological alterations studies, in the context of this neurodegenerative disease, have been performed through microscopic methodologies. The goal of the present work is to address if the modifications in the mitochondrial-shaping proteins occurring in this disorder have implications in other cellular pathways, which might constitute important pathways for the disease progression. To do so, we conducted a novel approach through a thorough exploration of the available proteomics-based studies in the context of Parkinson’s disease. The analysis provided insight into the altered biological pathways affected by changes in the expression of mitochondrial-shaping proteins via different bioinformatic tools. Unexpectedly, we observed that the mitochondrial-shaping proteins altered in the context of Parkinson’s disease are, in the vast majority, related to the organization of the mitochondrial cristae. Conversely, in the studies that have resorted to microscopy-based techniques, the most widely reported alteration in the context of this disorder is mitochondria fragmentation. Cristae membrane organization is pivotal for mitochondrial ATP production, and changes in their morphology have a direct impact on the organization and function of the oxidative phosphorylation (OXPHOS) complexes. To understand which biological processes are affected by the alteration of these proteins we analyzed the binding partners of the mitochondrial-shaping proteins that were found altered in Parkinson’s disease. We showed that the binding partners fall into seven different cellular components, which include mitochondria, proteasome, and endoplasmic reticulum (ER), amongst others. It is noteworthy that, by evaluating the biological process in which these modified proteins are involved, we showed that they are related to the production and metabolism of ATP, immune response, cytoskeleton alteration, and oxidative stress, amongst others. In summary, with our bioinformatics approach using the data on the modified proteins in Parkinson’s disease patients, we were able to relate the alteration of mitochondrial-shaping proteins to modifications of crucial cellular pathways affected in this disease.

## 1. Introduction

Mitochondria are pivotal organelles for several cellular functions, namely, the production of ATP through oxidative phosphorylation, the regulation of the Krebs cycle, fatty acid metabolism, gluconeogenesis, heme-synthesis, calcium and redox homeostasis, cell signaling, and the amplification of apoptosis [1]. They are highly dynamic organelles, as they can change their shape in response to cellular stimuli by fusion and fission processes and by their movement along the cellular cytoskeleton [2]. Alterations of mitochondria morphology can significantly influence several functions of the cellular metabolism, not only related to energy production but also in communication with the cytosol and the import and export of proteins, lipids, solutes, and metabolites or even the cytosol protection from possible harmful effects of certain mitochondrial components [3]. Mitochondria dynamic processes are of utmost importance for the mitochondrial growth rate, their redistribution within the cell, and for the maintenance of healthy mitochondria and proper functioning hence their alterations are frequently associated with different pathological conditions [4].

Parkinson’s disease is a highly debilitating condition, being a common neurodegenerative disease, and more than 10 million people worldwide are affected by this disease [5]. Currently, its etiology is not fully unraveled; however, evidences point to the importance of mitochondria in its pathobiology. Clinical features include mainly motor-based dysfunctions such as bradykinesia, resting tremor, or cogwheel rigidity [6]. Those features are a consequence of the loss of dopaminergic (DA) neurons in the substantia nigra (SN). The association between mitochondrial dysfunction and the pathobiology of Parkinson’s disease was first described in 1989. By using post mortem tissue from human patients, a functional deficiency was found on the mitochondrial Complex I from the respiratory chain [7,8]. Accumulating evidence shows the occurrence of mitochondria fragmentation in the context of different models of the pathology. Furthermore, alteration of the expression levels of different proteins linked to Parkinson´s disease (e.g., PINK1, Parkin, or DJ-1) are known to induce mitochondria fragmentation in DA neurons [9,10]. Recently, it was observed that alterations of the mitochondrial morphology can be related to their functional state and new tools were consequently designed to analyze mitochondrial shape and predict mitochondrial function [11].

Interestingly, several hypotheses for the specific loss of the DA neurons from the SN are also related to the vulnerability associated with the mitochondria of these neurons [12]. It has been suggested that DA neurons from the SN are more susceptible to oxidative stress due to the production of reactive oxygen species (ROS) during dopamine degradation, and these neurons present fewer amounts of antioxidants than other DA neurons within the brain [12]. They have very long axons in which mitochondria travel and also fragment to be able to accommodate within the synaptic terminals. Besides, it is known that DA neurons from the SN present lower mitochondria mass in the soma than in the dendrites, indicating that alterations of either fragmentation or movement along the cytoskeleton might have a bigger impact on these neurons [12]. Additionally, most of the substances that are used to model the disease directly target the mitochondria and induce the specific degeneration of the DA neurons.

In this study we made a thorough literature search to identify the mitochondrial proteins involved in controlling mitochondrial morphology that are differentially expressed in Parkinson’s disease. The altered biological pathways that might be affected by changes in the expression of these mitochondrial-shaping proteins in Parkinson’s disease were identified and analyzed. Considerations were made to better understand the biological mechanisms involved in this debilitating disease.

## 2. Methods

### Literature Search

For the compilation of the mitochondrial proteins involved in controlling mitochondrial morphology (Table 1), two independent users performed a search on PubMed, Science Direct, and Google up to 7 July 2017 using the following keywords in separate queries: “mitochondrial morphology”, “mitochondrial shape”, “mitochondrial organization”, “mitochondrial fusion”, “mitochondrial fission”, and “mitochondrial dynamics”. Only *Homo sapiens* proteins were gathered. The corresponding gene name and synonyms were collected in Table 1 by searching at the Universal Protein Resource (UniProt) databases [13].

To collect the information from proteomic-based studies of the differentially expressed proteins in the context of Parkinson’s disease, a search on PubMed and Web of Science (version v5.24) (up to 28 July 2017) was performed using the following keywords: “Parkinson’s disease mass spectrometry”, “Parkinson’s disease proteomics”. Studies working with samples from human patients and cellular models (from cell lines of human origin) were used to build the Supplementary Table S1. Three experienced reviewers selected the list of articles relevant for data extraction, taking into consideration only the studies that match the following criteria: proteomics studies, with information on differentially expressed proteins related to control conditions, employing samples either from human patients or cellular models (using cell lines of human origin).

Mitochondrial-shaping proteins were crossed with the proteins that have been found to be altered in Parkinson’s disease using the respective gene names in Venny web tool (v.2.1.0) [71]. The gene name of the common proteins (proteins that are involved in mitochondrial shape and are modified in Parkinson’s disease) were further used to determine the binding partners in HIPPIE web tool [72].

Network analysis was performed using the Cytoscape software (version v3.5.1) (Cytoscape Consortium, San Diego, CA, US)) with the plugins ClueGo (version v2.3.2) and Cluepedia (version v1.3.2). We used ClueGO’s default settings: merge redundant groups with >50.0% overlap; the minimum GO level used was 3 and the maximum GO level was 8; statistical test used was “Enrichment/Depletion (Two-sided hypergeometric test)”; Kappa Score Threshold was 0.4; and number of genes was set at 2 with a minimum percentage at 4.0.

## 3. Results

### 3.1. Differentially Expressed Mitochondrial Proteins Associated with Parkinson’s Disease

**Figure 1:**
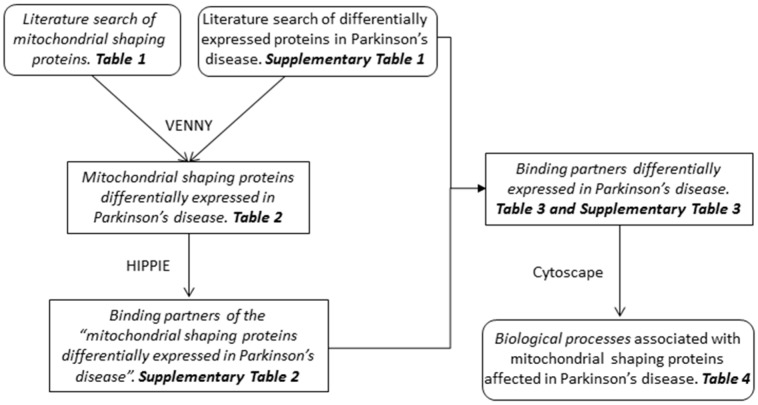
Flowchart showing the main steps used to identify the biological processes related to the mitochondrial-shaping proteins affected in Parkinson’s disease.

The interplay between mitochondria function and Parkinson’s disease was first described as a deficiency of the mitochondrial respiratory chain Complex I [7,8]. Alterations of the mitochondrial shape have been related to their functional state [11] and, in the past few years, an increasing number of reports have shown alterations of mitochondrial morphology in the context of Parkinson’s disease [73,74,75,76]. Mitochondrial morphology is tightly regulated by the combined action of proteins involved in fusion, fission, and movement along the cytoskeleton [3]. In this study we aimed to integrate the proteins related to mitochondria morphology with Parkinson’s disease pathology. The flowchart followed in the present work is represented in Figure 1. The complete list of mitochondrial proteins that have been described to play a role in the control and regulation of mitochondrial morphology is depicted in Table 1. To integrate the alterations of the mitochondrial dynamics in the context of the pathobiology of Parkinson’s disease, a literature search for proteomics-based studies in this disorder, that used samples from patients or cellular models (cell lines of human origin) (Supplementary Table S1), was performed. These proteins were then cross-referenced with the mitochondrial-shaping proteins listed in Table 1 (Figure 1).

**Figure 2:**
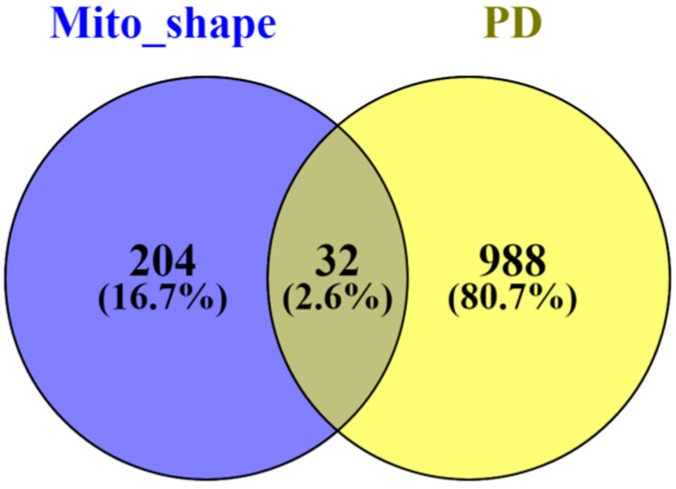
Venn diagram displaying the comparison of the number of the proteins found differentially expressed in the context of Parkinson’s disease (Supplementary Table S1) and the mitochondrial-shaping proteins described in the literature (Table 1). The Venn diagram was constructed using the Venny 2.1 software [71]. PD—Parkinson’s disease.

From this analysis, 32 different gene names (Figure 2), related to mitochondrial morphology, were found to be modified in the context of Parkinson’s disease, which correspond to 22 different proteins (Table 2). The vast majority of these mitochondrial proteins are related to the cristae morphology (82%), whereas only 9% are reported to be involved in the fusion and fission processes.

Curiously, in Parkinson’s disease the most reported mitochondrial morphology alterations are associated with mitochondrial fragmentation and movement impairment [74,77]. Mechanisms proposed for these alterations include: the alteration of the interaction between mitochondria and the motor complexes, and mitophagy impairment [78,79,80,81,82,83].

In our analysis, the fusion- and fission-related proteins found to be altered were Mitofusin-2 and the synaptic vesicle membrane protein VAT-1 homolog, as well as the dynamin-like protein (Drp1) and the SH3-containing protein SH3GLB2, respectively.

Although commonly accepted as a protein involved in mitochondrial fusion, the Mitofusin-2 protein also plays a key role in Ca^2+^ signaling. This function is facilitated by the physical interaction between the ER and the mitochondria for the delivery of Ca^2+^ to the mitochondrial matrix, enabling mitochondrial signaling. Hence, Mitofusin-2 is involved in both mitochondrial morphology and crosstalk between the ER and the mitochondria [84]. In the process of mitochondrial fusion both Mitofusin-1 and -2 have been reported to contribute, and although they share a common function in this process; deficiency in Mitofusion-2, but not Mitofusin-1, has been linked to neurodegenerative diseases [85]. Interestingly, the synaptic vesicle membrane protein VAT-1 homolog was found to be negatively regulate mitochondrial fusion in cooperation with Mitofusin-2 [70].

Regarding the mitochondrial fission process, in the context of Parkinson’s disease, the role of Drp1 has been extensively recognized in mitochondrial fragmentation in different animal and cellular models preceding neuronal death [73,86,87]. This large GTPase is a cytosolic protein that, following mitochondrial fragmentation stimuli, translocates to the outer mitochondrial membrane where it assembles into large complexes in a spiral form, enabling the constriction of the mitochondria [19]. Endophilins might be involved in membrane shaping, e.g., Endophilin B2, although they have also been described to play a role in mitophagy by promoting the degradation of the inner mitochondrial membrane [63].

Nevertheless, as reported above, most of the proteins related to mitochondrial morphology that we found to be altered in the context of Parkinson’s disease are associated with the regulation of the mitochondrial cristae morphology. Interestingly, accumulating evidence shows an association between the morphology of the mitochondrial cristae and the OXPHOS complexes. This fact brings forward the idea that the formation of the supercomplexes of the respiratory chain is related to the organization of the inner mitochondrial membrane [18]. The involvement of the mitochondria in Parkinson’s disease is clear, and several indications also reveal that alterations in the balance of fission and fusion processes increase the occurrence of fragmented mitochondria. However, the data collected in the present work points to a major contribution of the modification of the mitochondrial cristae. The major drawback when studying mitochondrial morphology is the fact that many studies have employed immunofluorescence using antibodies against mitochondrial membrane proteins and subsequent observation in optical microscopes. This methodology exhibits a lack of resolution needed to visualize the morphological subtleties in the mitochondrial cristae [88]. Therefore, for the study of mitochondrial dynamics, super-resolution and immunoelectron microscopy are better options since it is then possible to visualize the inner mitochondrial compartment [88,89].

The mitochondrial contact site and the cristae-organizing system (MICOS) have been described as a multiprotein complex relevant to inner membrane architecture [1,90]. In fact, it was described that some of the MICOS subunits control the morphology of the cristae in coordination with the mitochondrial Complexes III and IV from the respiratory chain [18]. In the absence of MICOS, cristae morphology is aberrant and these respiratory chain complexes are not functional [14]. Interestingly, in our study we found that two of the MICOS core subunits were altered in the context of Parkinson’s disease (Table 2): Mitofilin [18] and Mic26 [14]. The MICOS Complex interacts with proteins of the outer mitochondrial membrane, specifically with the sorting and assembly machinery component 50 (SAM50). Its depletion has been found to affect the mitochondria ultrastructure and the loss of cristae, thus affecting the assembly of the complexes of the mitochondrial respiratory chain [91]. Curiously, the SAM50 protein expression was also found to be altered in our analysis (Table 2).

Other modified proteins retrieved in our study included proteins from the mitochondrial respiratory chain such as members of the Complex III (the cytochrome b-c1 Complex subunit, the ubiquinol cytochrome c reductase iron–sulphur subunit, and the cytochrome b-c1 Complex subunit 6) and Complex IV (the cytochrome c oxidase subunit 4, the cytochrome c oxidase subunit 5A, the cytochrome c oxidase subunit 6C, the cytochrome c oxidase subunit 7C, and the cytochrome c oxidase polypeptide I).

Pivotal for the proper architecture of the mitochondrial cristae is the protein OPA1 [3]. Alterations were found to occur in the expression of the calcium-binding mitochondrial carrier protein Aralar2 (Slc25A), which acts in conjunction with OPA1 to sense modifications of the substrate levels for energy production. Following this interaction, the cristae are narrowed and the dimerization of the ATP synthase is stimulated [18].

The mitochondrial cristae structure is not solely maintained by proteins, but also by cardiolipin lipids [18], and several cardiolipin binding proteins have been described to be present at the mitochondrial membrane, amongst them the Prohibitin protein family. Prohibitin and Prohibitin-2 appeared to be altered in the context of Parkinson’s disease in our analysis (Table 2). These proteins are known to be organized in complexes within the inner mitochondrial membrane and are important for the proper organization of the cristae morphology and mitochondrial respiration [53,92]. Moreover, these proteins are involved in the turnover of the subunits of the mitochondrial respiratory chain and participate in the assembly of the Complex IV from the respiratory chain [93,94].

There is evidence that aberrant cristae morphology affects the proper assembly of the OXPHOS complexes, but also that the lack of ATP synthase subunits impacts the morphology of the cristae [95,96]. The latter has been supported by studies showing that ATP synthase dimerization forces membrane curvature [97]. Interestingly, mitochondrial morphology also relies on the cellular energetic state since, by compromising mitochondrial membrane potential, mitochondrial fragmentation is induced [98]. In addition, dimer formation of the F1F0-ATP synthase affects mitochondrial cristae structure [95].

### 3.2. Binding Partners of Mitochondrial Proteins Differentially Expressed in Parkinson’s Disease

**Figure 3:**
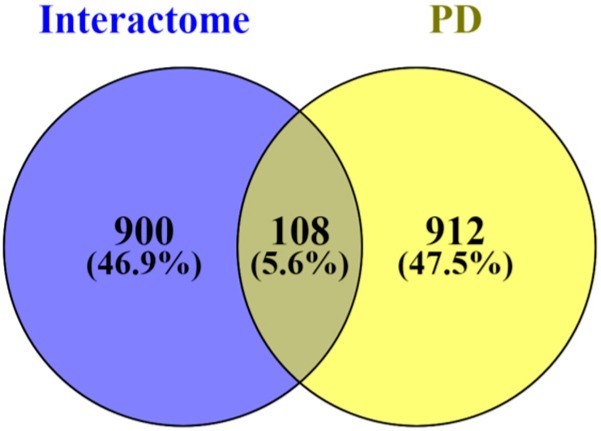
Venn diagram displaying the comparison of the number of the binding partners of the mitochondrial-shaping proteins affected in Parkinson’s disease (Table 2) and the proteins found to be differentially expressed in the context of Parkinson’s disease (Supplementary Table S1). The Venn diagram was constructed using the Venny 2.1 software [71]. PD—Parkinson’s disease.

Mitochondria are organelles with important roles in many cellular processes, hence we next explored the binding partners described for the mitochondrial proteins differentially expressed in Parkinson’s disease (Table 2). Using the HIPPIE tool [72,99,100,101], we determined the complete list of binding partners, which is depicted in Supplementary Table S2 (Figure 1). This software provides information on human protein-protein interactions with high confidence scores that are due to the amount of supporting data available as well as derived from annotated information [72,99]. In total, for the 22 mitochondrial-shaping proteins altered in Parkinson’s disease, we found 1683 hints of interacting proteins. Since some of the mitochondrial-shaping proteins have interacting proteins in common, these hints correspond to 1008 different proteins. When we cross-referenced these binding partners with the list of proteins found to be modified in the context of Parkinson’s disease (Supplementary Table S1), 108 common hints were identified (Figure 3, Table 3, and Supplementary Table S3). In Supplementary Table S3, the different hints of the proteins listed in Table 3 within the different proteomics-based studies used in the present work are described.

**Figure 4:**
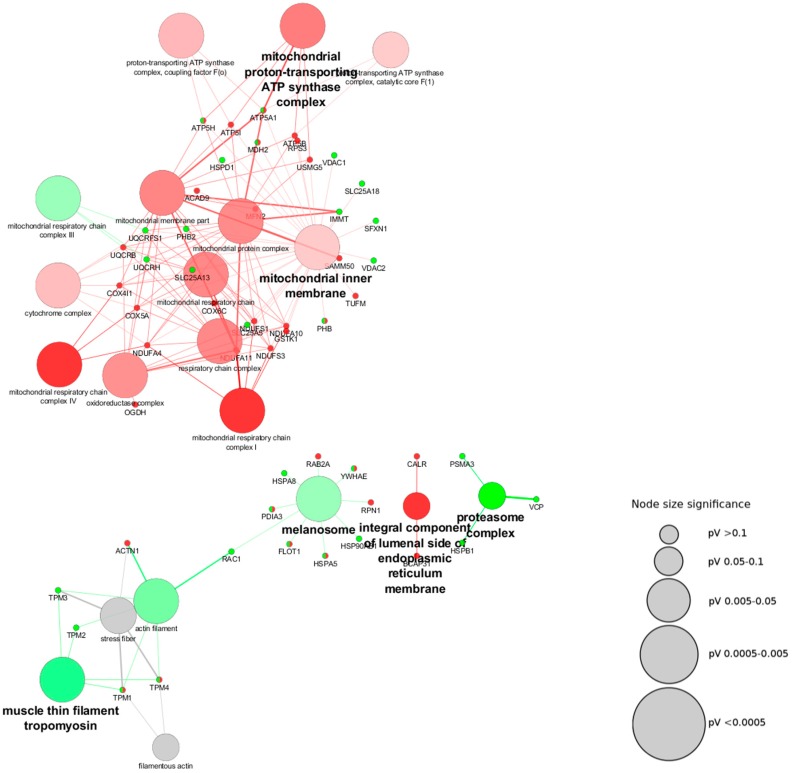
Cytoscape network of the main cellular components associated with mitochondrial-shaping proteins in the context of Parkinson’s disease. The binding partners of the mitochondrial-shaping proteins found to be modified in different proteomics-based approaches in the context of Parkinson’s disease according to the Human Integrated Protein-Protein Interaction rEference (HIPPIE) and appearing as modified in proteomics-based studies on Parkinson’s disease were subjected to network analysis using the plugin ClueGo from the software Cytoscape to analyze the cellular components represented by the proteins from the list. Gray scale nodes correspond to cellular components that were found to be equally up- and downregulated in different studies, while green and red nodes are representative of upregulated and downregulated cellular components respectively, in the context of Parkinson’s disease. The increase in green and red color gradient represents higher amounts of the contribution of up- and downregulated proteins, respectively. The size of the nodes is indicative of their statistical significance.

As a first approach we assessed the cellular components, which are represented by these proteins using the plugin ClueGO in Cytoscape software (Figure 1). In Figure 4 it is possible to see the network built from the different cellular components and their upregulation (green nodes) or downregulation (red nodes) in Parkinson’s disease. The cluster of proteins that were found to be upregulated fall into diverse cellular components: mitochondrial respiratory chain Complex III, proteasome complex, muscle thin filament tropomyosin, and melanosome. On the other hand, the downregulated are represented by: inner mitochondrial membrane, integral component of the lumenal side of the endoplasmic reticulum membrane, and mitochondrial proton-transporting ATP synthase complex.

The proteins used to search for the binding partners were mitochondrial proteins, thus alterations in the mitochondrial cellular components were expected. In this regard, the downregulation of the inner mitochondrial membrane and the mitochondrial proton-transporting ATP synthase complex was anticipated. Several of the proteins found to be altered in the context of Parkinson’s disease (Table 2) are proteins located at the inner mitochondrial membrane, and the alteration of these components has also previously been described in the pathology [7].

Interestingly, it has been shown that the ubiquitin-proteasome system regulates the level of proteins targeted to the mitochondrial intermembrane space, and this process depends on the mitochondrial intermembrane space import machinery [102]. Additionally, it has been shown that the ubiquitin-proteasome system acts on the regulation of the mitochondrial biogenesis [103]. In this study, we found an upregulation of the proteasome, which corroborates the evidence of its dysfunction in Parkinson’s disease [103,104]. ROS levels are increased in Parkinson’s disease and are responsible for the oxidative modification of lipids, DNA, and proteins [105]. These modifications might lead to misfolded proteins and aggregation [106]. Mitochondrial proteins might be dysfunctional due to the harmful effects of ROS, which not only might modify the folded proteins, but also affect the incorporation of newly synthetized mitochondrial proteins since they are translated in the cytosol and must be transported unfolded into the mitochondria [103]. In an oxidative stress scenario, as in Parkinson’s disease, the risk of the alteration of unfolded proteins and consequent removal by the proteasome is higher, reducing the amount of mitochondrial proteins available. Besides, it is known that outer mitochondrial membrane proteins involved in mitochondrial fusion are regulated by ubiquitination and that this process is induced by stress [19].

Mitochondrial dynamics not only relies on mitochondrial fusion and fission proteins, but also on the contact sites between mitochondria and the ER, which are fundamental for the initial fission process [107]. It has been described that the shape-forming proteins control mitochondrial morphology by mediating the attachment of the mitochondria to the cytoskeleton and the ER [107,108], and they can also connect the inner and outer mitochondrial membranes, hence influencing the import and assembly of mitochondrial proteins [109]. Regarding the upregulation of the melanosome as a cellular component, although it is an organelle not present in neuronal cells, when we look closely at the proteins contributing to this node, we find that three of the proteins are heat shock proteins. These proteins are key components in ensuring proper protein function and are expressed in response to stress, controlling the subsequent degradation of misfolded proteins, which is also in line with the upregulation of the proteasome complex and the occurrence of the oxidative stress characteristic of the disorder.

The mitochondrial cytochrome bc1 complex from the respiratory chain (Complex III) is one of the main producers of ROS, together with the Complex I [110]. Although Complex I release superoxide into the mitochondrial matrix, Complex III does it into the intermembrane space and the cytosol [111]. In the pathobiology of Parkinson’s disease, it is well accepted that there is an increase in ROS leading to oxidative stress [105], which is in agreement with the upregulation of the cellular mitochondria component of the respiratory chain Complex III found in our analysis. This complex is localized in the inner mitochondrial membrane, at the cristae, and has three transmembrane subunits in which the prosthetic groups involved in the redox reactions are located. They must be dimerized for proper functioning, which is also dependent on the mitochondrial membrane potential [110], suggesting that alterations in the organization of the inner mitochondrial membrane might affect their function.

A closer look at the network shows that “muscle thin filament tropomyosin” is connected to cellular components related to the actin cytoskeleton (“actin filament”, “stress fiber”, and “filamentous actin”). As described above, the cytoskeleton also plays a role in the dynamics and movement of the mitochondria [108]. Interestingly, in the context of Parkinson’s disease some models (both genetic and drug-based) showed a negative impact on the dynamics of the actin cytoskeleton and the formation of stress fibers [108,112,113].

### 3.3. Biological Processes Associated with Mitochondrial-Shaping Proteins Affected in Parkinson’s Disease

**Figure 5:**
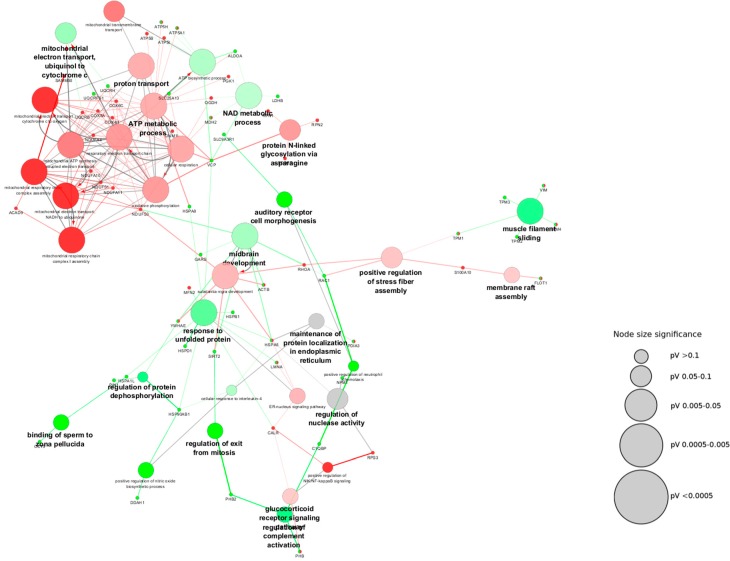
Cytoscape network of the main biological processes associated with mitochondrial-shaping proteins in the context of Parkinson’s disease. The binding partners of the mitochondrial-shaping proteins found modified in different proteomics-based approaches in the context of Parkinson’s disease were subjected to network analysis using the plugin ClueGo from the software Cytoscape to analyze the biological processes affected in the disease. Gray scale nodes correspond to biological pathways that were found to be equally up- and downregulated in different studies, while green and red nodes are representative of upregulated and downregulated biological pathways, respectively, in the context of Parkinson’s disease. The increase in green and red color gradient represents higher amounts of the contribution of up- and downregulated proteins, respectively. The size of the nodes is indicative of their statistical significance.

To obtain information on the biological processes related to the mitochondrial-shaping proteins affected in Parkinson’s disease, we undertook a bioinformatic approach using the plugin ClueGo from the Cystoscope software (Figure 5 and Table 4). This plugin allows the extraction of the biological meaning of large lists of proteins [114]. Overall, around 44% altered processes are related to energy production by the mitochondria. This contribution was expected since the dysfunction of this organelle is a hallmark of the disease.

Interestingly, other biological processes are related to the occurrence of oxidative stress and the respective alterations in proteins, which is also a known characteristic of Parkinson’s disease [105]. In this regard, the positive regulation of the nitric oxide (NO) biosynthetic process have been shown to occur in this disorder, which is relevant for neuronal death. When NO synthases are ablated, animals are protected against the effect of the MPTP toxin [115]. It is also known that NO not only induces oxidative stress but also neuronal death [116,117]. The downregulation of the glutathione derivative biosynthetic process is also a characteristic of an oxidative stress scenario, which has also been described in Parkinson’s disease [118]. This peptide acts as a cellular antioxidant, which is produced by neurons and glial cells, and it has been proposed as an important molecule for therapeutic purposes in the context of Parkinson’s disease [118,119]. Moreover, the upregulation of the response to unfolded proteins is important in an oxidative stress scenario where proteins and peptides can be oxidatively modified with a harmful effect on their three-dimensional (3D) structure, with aggregation having a negative impact on their function [106]. Besides, protein oxidative modifications and aggregation have been also related to the decreased in glutathione levels [120].

As stated along this work, the cellular cytoskeleton is one of the mechanisms contributing to the definition of mitochondrial morphology [108]. In fact, modification of the actin cytoskeleton has been probed in Parkinson’s disease [108,112,113]. Mitochondrial fusion and fission processes are affected by the interaction of the mitochondria with the cytoskeleton. It has been described that the fusion process can be delayed when actin filaments are depolymerized [121]. The actin cytoskeleton is also involved in the fission process [38]. In our network, several processes are related to the actin cytoskeleton, such as the upregulation of the process of “muscle filament sliding” and the downregulation of the “positive regulation of stress fiber assembly”. Interestingly, after a closer look at the modified biological process of “binding of sperm to zona pellucida”, we observed that the proteins connected to this process are molecular chaperones and, remarkably, TCP-1-epsilon is known to play a role in the folding of actin and tubulin [122].

Regarding the “auditory receptor cell morphogenesis” biological processes, it is important to note that the protein Rac1 contributes to this pathway and is involved in the regulation of secretory processes, the phagocytosis of death cells, cell polarization, and the formation of membrane ruffles. In the context of Parkinson’s disease, it has been shown to contribute to a ROS generating pathway acting with Nox1, causing neuronal death [123]. Interestingly, the other component of this node is the NHERF-1 protein, which has been shown to act as a scaffold for connecting plasma membrane proteins with members of the ERM (ezrin/moesin/radixin) family, aiding in their link to the actin cytoskeleton for the regulation of their surface expression [124].

Within the highlighted process of “regulation of protein dephosphorylation” involved in the regulation of protein function, we found an interesting protein contribution to this pathway: the Peptidyl-prolyl cis-trans isomerase (Pin1). This protein has been shown to be involved in the disease, being upregulated in cellular and animal models as well as in SN in patients [125]. The alteration of this biological process might have an impact on stress responses, immune function, and neuronal survival [126]. Also, this pathway is fundamental for proper mitochondrial functioning and signaling since, in response to the metabolic state of the cell, mitochondrial proteins from the import machinery might be regulated by phosphorylation [127].

In the “regulation of exit from mitosis” process, two interesting proteins emerge: Prohibitin-2 and the NAD-dependent protein deacetylase sirtuin-2. The latter deacetylates lysines on histones, alpha-tubulin, and other proteins [128]. By acting on tubulin it has a direct impact on microtubule function. Tubulin can be subjected to different post-translational modifications with influence on the microtubule polymerization state and its function, such as acetylation [129]. This modification on the residue K40 has been reported to alter the interaction of proteins with the cytoskeleton, with subsequent impact on the intracellular transport along the microtubules [129]. As described in this work, this may affect mitochondria morphology and dynamics.

Parkinson’s disease has long been linked to increased inflammatory response [130]. In our analysis we found that the processes related to the inflammatory response were upregulated: “positive regulation of neutrophil chemotaxis” and “regulation of complement activation”. Interestingly, from the last process, two of the implicated proteins belong to the Prohibitin family, which have been described to be involved in the regulation of mitochondrial respiration [131]. Regarding the downregulation of the process of the “glucocorticoid receptor signaling pathway” it is important to note that a decrease in the levels of the glucocorticoid receptor in both the SN of patients and in animal models of the disease has been reported [132,133]. These receptors regulate inflammation and are dysregulated in microglia in the context of Parkinson’s disease. Dysregulation has been proposed to sustain the chronic inflammatory state observed in this disorder as well as the increased permeability of the blood brain barrier, which might increase neuronal vulnerability [132,133]. Another pathway related to the inflammatory process is downregulated: “positive regulation of NF-kB signaling”, in which the protein ribosomal protein S3 stands out. Interestingly, this protein has been proposed to protect the dopaminergic neurons from apoptosis [134].

Within the network, the biological processes of “midbrain development” and “substantia nigra development” share three proteins: Actin, Complex I 30 kD from the mitochondrial respiratory chain, and the 14-3-3 protein epsilon. As described previously, the dysfunction of Complex I from the mitochondrial respiratory chain was the first indication of the mitochondria involvement in the pathobiology of Parkinson’s disease [7,8]. The contribution of the cytoskeleton has also been shown, in which actin has a key role in the secretion of the synaptic vesicles content that might be then translated into a decrease in the content of neurotransmitters in the synaptic cleft [135]. Although the 14-3-3 protein is ubiquitously expressed and participates in the regulation of many signaling pathways, it has also been found to be a constituent of the Lewy bodies of Parkinson’s disease patients [136].

In Parkinson’s disease there is a critical modification in the lipid rafts composition, and increasing evidence shows their contribution to the disorder [137,138,139]. Lipid rafts have a role in diverse cellular processes such as membrane trafficking, signal transduction, and cytoskeletal organization. Their alteration can also have a negative impact on protein-protein interactions, which are fundamental processes for the formation of protein supercomplexes [138]. Interestingly, we found the process of “membrane raft assembly” to be downregulated. Not only was the protein Flotillin-1, a well-known component of the lipid rafts, found to contribute to this node, but also the protein S100A10. The S100 family of proteins are involved in several cellular processes such as the regulation of cell proliferation and differentiation, apoptosis, calcium homeostasis, energy metabolism, and inflammation. Interestingly, they also interact with cytoskeletal and other cellular proteins [140]. Some of the membrane proteins that interact with the S100A10 are: Annexin 2, ion channels, actin binding proteins, and the serotonin receptor [140]. This protein has been proposed to function on membrane repair and was shown to be downregulated in depressive-like states in mice, with its expression being regulated by neurotrophins [141,142].

*N*-glycosylation is a post-translational modification that is found in membrane proteins and secreted proteins; amongst them are growth factors and their receptors [143]. In our analysis, we found that the process of “protein N-linked glycosylation via asparagine” was downregulated. This modification takes places in the ER and in the Golgi, having an effect on protein function. Evidence show that *N*-glycosylation is important for proper neuronal function and has a role in synaptic transmission [144], hence having a profound impact on the disease [145].

The occurrence of a role of the ER stress in the context of Parkinson’s disease [146] is supported through new evidence, and the process of the “ER-nucleus signaling pathway” was found to be downregulated in our analysis. In this node, we found the LMNA. Lamins are filamentous proteins that contribute to the nucleus architecture and gene expression [147,148]. These proteins also interact with the actin cytoskeleton, which is known to be affected in Parkinson’s disease [108,112,113,149]. The other proteins (calreticulin and the heat shock 70 kDa protein 5) are chaperones involved in protein folding and the formation of multimeric complexes [150,151], playing a crucial role in an oxidative stress scenario. Additionally, in the case of fission, not only actin but also the ER is involved in the process of mitochondrial preconstruction and DRP1 assembly [107].

## 4. Discussion

Mitochondria are fundamental organelles for cells, working mainly on energy production, calcium homeostasis, and apoptosis. Defects in the mitochondrial respiratory chain have received much of the attention as a key player in the pathobiology of Parkinson’s disease [8,152]. However, additional modifications of the mitochondria are being increasingly reported [153,154]. Besides, it is now known that the inhibition of Complex I from the mitochondrial respiratory chain by MPP+ and rotenone does not directly trigger cytochrome c release but, instead, increases the amount of cytochrome c within the mitochondrial intermembrane space [155], indicating that there are other processes required to trigger neuronal death. Amongst them are changes in mitochondrial dynamics (i.e., alterations in the fusion and fission processes, alteration of cristae morphology) [85,156].

Increasing evidence shows that for proper mitochondrial function, processes like mitochondrial fusion, fission, and turnover are fundamental, and their dysfunction has been linked to different diseases [1,3]. In the context of Parkinson’s disease, an increase in mitochondrial fission has been reported [73,157,158], suggesting that this excessive fragmentation might then enhance cytochrome c release from mitochondria and subsequently triggering apoptosis [159]. However, we found the process of cristae remodeling to be more highlighted in our analysis regarding the alteration of the mitochondrial-shaping proteins in Parkinson’s disease. Notably, the alteration of the mitochondria cristae and membrane might affect the proper binding of cytochrome c, favoring its release to the cytosol and initiating the apoptotic process [155]. Some evidence of mitochondrial cristae remodeling exists in the context of Parkinson’s disease. One study using a cybrid cell line constructed with mitochondria DNA isolated from cells from Parkinson’s disease patients showed that there were deficiencies in both complexes of the mitochondrial respiratory chain I and IV, and cells contained a non-homogenous mitochondrial population with different morphologies ranging from enlarged to swollen and rounded in shape, which also displayed different mitochondrial membrane potential values [160]. At the ultrastructural level, some mitochondria from this cybrid displayed a decreased in the matrix density and contained a reduced number of cristae and a discontinuous outer mitochondrial membrane [160]. Similarly, in a study using transgenic mice for mutated α-synuclein, morphological alterations on the cristae were also observed, showing a disordered inner membrane and swollen matrix [34]. However, this type of study with samples from human patients is insufficient, and an increasing number of studies aiming at deciphering the ultrastructure of the mitochondria in Parkinson’s disease by electron and super-resolution microscopy are required.

It is known that cristae remodeling is fundamental during apoptosis for the proper release of cytochrome c [27], and that the cristae are the sites were the OXPHOS components are located (94% of Complex II and ATP synthase [161], and 85% of cytochrome c [27]). Compelling data indicate that the shape of the cristae is crucial for the modulation of the OXPHOS function [18], and relies on the cellular state [162,163]. Disruption of the cristae junctions is a result of the release of apoptotic factors from the mitochondria [27]. Remarkably, in our analysis most of the proteins involved in mitochondrial dynamics that were found to be altered in the context of Parkinson’s disease have been previously reported to play a role in the morphology of the cristae.

When imaging mitochondria, four main components can be distinguished at the ultrastructure level: the outer mitochondrial membrane (OMM), important for regulating membrane permeability and the import/export of mitochondrial proteins; the inner mitochondrial membrane (IMM), where the mitochondrial respiratory chain is placed (the invaginations of the IMM into the matrix are the so-called mitochondria cristae); the intermembrane space (IMS), which is the space between the two mitochondrial membranes; and the matrix, where the components of the tricarboxylic acid (TCA) cycle are located. The inner mitochondrial membrane is organized into three specialized zones: the inner boundary membrane, where the inner and outer membranes are associated, containing proteins of the protein import machinery; the cristae, which are the inner membrane invaginations that are enriched in proteins involved in protein translocation and synthesis as well as proteins involved in iron-sulfur biogenesis; and the cristae junctions, which are the portion of the cristae that are constricted and where the MICOS Complex is located [3].

Fundamental to cell survival is the implication of the mitochondria in the regulation of apoptosis. Within this organelle several pro-apoptotic proteins reside, triggering the apoptotic process when released into the cytosol. The permeabilization of the OMM constitutes a point of no return in the activation of this process, where the Bcl2 family of proteins participates in its regulation [164]. Interestingly, our network analysis highlighted the alteration on the permeability of membranes, supporting the apoptotic activation [165].

The OMM morphology is influenced by its interaction with the ER, ribosomes, the nucleus, and the cellular cytoskeleton [3]. Fundamental to the regulation of multiple cellular processes are the mitochondrial-ER contact sites [164]. In our network analysis, this intraorganellar interaction was highlighted as being altered in Parkinson’s disease. Amongst the cellular processes are: the regulation of the intracellular calcium levels, mitochondrial fission, the endowment of membranes to phagosomes, and the formation of the inflammasome [166]. It is now clear how the ER participates in the initiation of the mitochondrial fission process. The ER enwraps the mitochondria at the constriction site where the dynamin-like protein Drp1 responsible for the fission process localizes [107,167]. Additionally, actin polymerization and the ER protein inverted formin 2 (INF2) are involved in this process [38]. Interestingly, actin filaments polymerized around the constriction sites might constitute the pulling force for the fission process [38], and several processes related to the actin cytoskeleton were found to be altered in our analysis. Additionally, the actin cytoskeleton plays a fundamental role in synaptic vesicle secretion. The alteration of this process affects synaptic transmission in the disease. Not only actin but also calcium is involved in this process.

The regulation of calcium levels is also dependent on the mitochondria-ER crosstalk, and a modification of its homeostasis has been reported in Parkinson’s disease [168]. Mitochondrial calcium channels display low affinity for this ion, and for correct calcium entry into the mitochondria for the formation of the ER-mitochondrial contact sites is fundamental [169]. The relevance of this interaction has been shown by its involvement in the progression of Alzheimer’s disease, where it is upregulated [170]. When calcium accumulates within the mitochondria, oxidative phosphorylation and ATP production are enhanced [171]. In addition, different chaperones are involved in the stabilization of these contact sites and could coordinate signaling between mitochondria and the ER [166]. In this direction, we also found that the expression of the chaperone grp78 was altered [172]. The ER-mitochondria contact sites are also related with the ER-stress response, which might trigger apoptosis [173]. This pathway has also been shown to be active in the context of Parkinson’s disease [174]. Additionally, the calcium released by the ER at these contact sites might act as an amplifier of the apoptotic pathway [166]. Besides, the fission protein Fis1 has also been shown to facilitate the cleavage of the pro-apoptotic protein Bap31 [175]. Interestingly, these contact sites are not only relevant to the regulation of calcium levels, but also to ROS-mediating signaling [176].

Moreover, the ER contacts with the phagosomes and non-functional mitochondria degraded by mitophagy are recognized by specific OMM proteins [166]. MFN2 has also been indicated as critical to autophagosome formation, and the ER-mitochondria interaction is important for autophagosome formation [166,177]. Accumulating evidence shows that the ER might also be involved in the mitochondrial fusion process, since it was shown that mitofusin2 (MFN2) is necessary for the tethering of both organelles [178].

Relevant to proper neuronal function is the appropriate localization of the mitochondria within the synaptic terminals, where they can provide ATP for exocytosis and regulate calcium levels during synaptic transmission [179]. Mitochondria positioning within these terminals relies not only on mitochondrial fission [180], since only small mitochondria might fit into the terminals, but also in correct mitochondrial movement along microtubules and the actin cytoskeleton [164]. In this work, the network analysis of cellular components and biological pathways indicated that in the context of Parkinson’s disease actin filaments are affected [108,112,113]. In addition, dysregulation of the cellular microtubules has been reported, namely in the alteration of proteins involved in tubulin acetylation. Most of the drugs used to induce Parkinson’s disease modify microtubules, and specifically, acetylation has been shown to affect the interaction of proteins with the cytoskeleton [129]. Alteration of the cellular cytoskeleton might have an impact not only on mitochondrial fission but also distribution within neurons [181,182,183,184]. The modification of the actin cytoskeleton might also have an impact on the regulation of surface receptor distribution [124], which was highlighted in the present work.

The ATP produced by the mitochondria reaches the cytosol by its active transport through the adenine nucleotide translocator. To produce ATP, the processes of the TCA cycle and the respiratory chain/oxidative phosphorylation system act in conjunction. The last is located at the IMM. As a result of the mitochondrial respiratory chain deficiency, ATP production is reduced and ROS are increased, which leads to oxidative stress. This increase leads to modifications in protein, lipids, and DNA within the cells [185]. Amongst the modified lipids, the oxidation of cardiolipin from the IMM has been reported in the context of Parkinson’s disease [155], and its oxidation disrupts the normal binding of cytochrome c to the membrane [155]. Furthermore, ER is fundamental for providing membrane lipids to the mitochondria [186], highlighting again the importance of this crosstalk. Phosphatidic acid is considered a fusogenic lipid required for the fusion mediated by mitofusins [187]. Cardiolipin have been reported to control mitochondrial fission [188,189]. Interestingly, in the present work we found the expression of several cardiolipin binding proteins to be modified, which might impact the proper assembly of the mitochondrial membranes. Moreover, synaptic mitochondria present lower levels of cardiolipin, which has been pointed out as a lower threshold for the release of cytochrome c in the apoptotic process [164,190]. It has also been reported that synaptic mitochondria present higher sensitivity to the inhibition of the mitochondrial respiratory chain Complex I [191]. Detachment of cytochrome c from the membrane is necessary for cytochrome c release for apoptotic activation and cristae remodeling [27]. As discussed above, these fundamental processes have been emphasized by the high percentage of mitochondrial-shaping proteins found to be altered in the context of Parkinson’s disease in contrast with the proteins involved in fusion and fission.

The ER-mitochondrial connection is also important for the inflammatory response [192]. The activation of the inflammasome might occur under an oxidative stress scenario, i.e., Parkinson’s disease, where there is an increase in ROS production by the mitochondria [192]. Specific receptors are translocated to the ER-mitochondrial contact sites in response to inflammation [192]. Besides, relevant to the activation of this inflammasome is the VDAC channel located at the mitochondria. Knockdown of both VDAC1 and -2 abolishes the inflammasome formation [192]. Both channels were found to be downregulated in our study. Furthermore, these channels interact with Bcl-2 proteins, therefore enabling cell survival [192,193].

## 5. Conclusions

The DA neuron loss from the SN constitutes a hallmark of Parkinson’s disease. These neurons are known to be more susceptible than other DA neurons in the brain, and some of the referred sources for this vulnerability are associated with mitochondria. A more complex picture of the alterations of the mitochondria in Parkinson’s disease is arising in addition to the widely known deficit in the mitochondrial Complex I dysfunction. Through the so-called “mitochondrial life cycle”, these organelles can modulate their function and perform quality control. Accumulating evidence shows that there is a correlation between the morphology of these organelles and the cellular energy status. Increasing efforts have been made to associate the morphology of the mitochondria to its function. Importantly for a neurodegenerative disease such as Parkinson’s disease, in which the causes have been linked to mitochondrial dysfunction, this type of analysis will aid in advancing the field, both in the pathobiology of the disease and the search for new therapies.

By network analysis we have correlated the changes in differentially expressed mitochondrial-shaping proteins in the context of Parkinson’s disease with the corresponding biological pathways affected in the disease. One of the most striking findings is related to the process of cristae remodeling, since most of the mitochondrial-shaping proteins found to be altered in the context of Parkinson’s disease participate in the maintenance of cristae shape. Remarkably, this alteration is evident in different human disorders, including Parkinson’s disease. Since these structures regulate protein and lipid distribution as well as soluble molecules (i.e., ADP and cytochrome c), their alteration might have a direct impact on neuronal physiology and survival.

In our opinion, although it is clear in the cellular and animal models of the disease that mitochondrial morphology is altered, more studies from post mortem tissue from patients are needed, aiming at unravelling the alterations of the mitochondrial morphology more specifically related to the cristae shape in the context of Parkinson’s disease. These studies would provide new insights into the development of new therapies or aid in biomarkers discovery. Identification of the mitochondrial components that play a role in the process of cristae remodeling might also be fundamental for these purposes.

## Figures and Tables

**Table 1:** List of mitochondrial-shaping proteins.

Gene Name (with Synonyms)	Protein Name	Function	Localization	Shaping Function	References
APOO, FAM121B, MIC23, MIC26, My025, UNQ1866/PRO4302	MICOS Complex subunit MIC26 (Apolipoprotein O) (MICOS Complex subunit MIC23) (Protein FAM121B)	Component of the MICOS Complex, a large protein Complex of the inner mitochondrial membrane that plays crucial roles in the maintenance of crista junctions, inner membrane architecture, and formation of contact sites to the outer membrane	IMM	Cristae shape	[14,15,16]
APOOL, CXorf33, FAM121A, MIC27, UNQ8193/PRO23204	MICOS Complex subunit MIC27 (Apolipoprotein O-like) (Protein FAM121A)	Component of the MICOS Complex, a large protein Complex of the inner mitochondrial membrane that plays crucial roles in the maintenance of crista junctions, inner membrane architecture, and formation of contact sites to the outer membrane	IMM	Cristae shape	[14,15,17,18]
ATP5A1, ATP5B, ATP5C1, ATP5D, ATP5E, ATP5F1, ATP5G1, ATP5G2, ATP5G3, ATP5H, ATP5I, ATP5J, ATP5J2, ATP5L, ATP5O, MT-ATP6, MT-ATP8	ATP synthase	ATP production	IMM	Cristae shape	[3,15,19]
ATPIF1, ATPI	ATPase inhibitor, mitochondrial (Inhibitor of F(1)F(o)-ATPase) (IF(1)) (IF1)	ATP production regulation	Matrix	Cristae shape	[3,20]
BAK1, BAK, BCL2L7, CDN1	Bcl-2 homologous antagonist/killer (Apoptosis regulator BAK)	Promotes apoptosis	OMM	OMM permeabilization	[3,21]
BAX, BCL2L4	Apoptosis regulator BAX	Accelerates apoptosis	OMM	OMM permeabilization	[3,19,21,22]
BCL2	Apoptosis regulator Bcl-2	Promotes cell survival	OMM	OMM permeabilization	[3,22]
BCL2A1, BCL2L5, BFL1, GRS, HBPA1	Bcl-2-related protein A1 (A1-A) (Hemopoietic-specific early response protein) (Protein BFL-1)	Promotes cell survival	OMM	Not clear function	[23,24]
BCL2L11, BIM	Bcl-2-like protein 11 (Bcl2-L-11) (Bcl2-interacting mediator of cell death)	Induces apoptosis and anoikis	IMM	Cristae remodeling	[25,26]
BID	BH3-interacting domain death agonist	The major proteolytic product p15 BID allows the release of cytochrome c	IMM	Cristae remodeling	[25,27]
BIK, NBK	Bcl-2-interacting killer (Apoptosis inducer NBK) (BIP1) (BP4)	Promotes apoptosis	IMM	Cristae remodeling	[25,28]
CHCHD3, MIC19, MINOS3	MICOS Complex subunit MIC19 (Coiled-coil-helix-coiled-coil-helix domain-containing protein 3)	Component of the MICOS Complex, a large protein Complex of the inner mitochondrial membrane that plays crucial roles in the maintenance of crista junctions, inner membrane architecture, and formation of contact sites to the outer membrane	IMM	Cristae shape	[14,16,18]
COA3, CCDC56, MITRAC12, HSPC009	Cytochrome c oxidase assembly factor 3 homolog, mitochondrial (Coiled-coil domain-containing protein 56) (Mitochondrial translation regulation assembly intermediate of cytochrome c oxidase protein of 12 kDa)	Core component of the MITRAC (mitochondrial translation regulation assembly intermediate of cytochrome c oxidase) Complex, which regulates cytochrome c oxidase assembly.	IMM	Cytochrome c oxidase	[29,30]
COX4I1, COX4I2, COX5A, COX5B, COX6A1, COX6A2, COX6B1, COX6B2, COX6C, COX7A1, COX7A2, COX7B, COX7B2, COX7C, COX8A, COX8C, MT-CO1, MT-CO2, MT-CO3	Mitochondrial Complex IV: cytochrome c oxidase subunits	ATP production	IMM	Cristae shape	[18]
CYC1, MT-CYB, UQCR10, UQCR11, UQCRB, UQCRC1, UQCRC2, UQCRFS1, UQCRH, UQCRQ	Mitochondrial Complex III: ubiquinol-cytochrome c reductase Complex subunits (UQCR)	ATP production	IMM	Cristae shape	[18]
DNAJC19, TIM14, TIMM14	Mitochondrial import inner membrane translocase subunit TIM14 (DnaJ homolog subfamily C member 19)	Probable component of the PAM Complex, a Complex required for the translocation of transit peptide-containing proteins from the inner membrane into the mitochondrial matrix in an ATP-dependent manner	IMM	Crista shape	[15,31,32]
DNM1L, DLP1, DRP1	Dynamin-1-like protein (EC 3.6.5.5) (Dnm1p/Vps1p-like protein) (DVLP) (Dynamin family member proline-rich carboxyl-terminal domain less) (Dymple) (Dynamin-like protein) (Dynamin-like protein 4) (Dynamin-like protein IV) (HdynIV) (Dynamin-related protein 1)	Mitochondrial and peroxisome division	OMM and cytosol	Fission	[3,18,22]
DNM2, DYN2	Dynamin-2 (EC 3.6.5.5)	Microtubule-associated force-producing protein involved in producing microtubule bundles and able to bind and hydrolyze GTP	Cytosol	Fission	[4,33]
FIS1, TTC11, CGI-135	Mitochondrial fission 1 protein (FIS1 homolog) (hFis1) (Tetratricopeptide repeat protein 11) (TPR repeat protein 11)	Mitochondrial fragmentation	OMM	Fission	[3,19,22]
FUNDC1	FUN14 domain-containing protein 1FUN14 domain-containing protein 1	Mitophagy	OMM	Fission	[29,34,35,36]
GDAP1	Ganglioside-induced differentiation-associated protein 1	Mitochondrial fission	OMM	Fission	[1,19,22]
hfzo1	Mitochondrial transmembrane GTPase Fzo-1	FUNDC1 mediates highly selective mitochondrial clearance under hypoxic conditions without impacting general autophagy	OMM	Fusion	[19]
IMMT, HMP, MIC60, MINOS2, PIG4, PIG52	MICOS Complex subunit MIC60(Cell proliferation-inducing gene 4/52 protein) (Inner mitochondrial membrane protein) (Mitofilin) (p87/89)	Component of the MICOS Complex, a large protein Complex of the inner mitochondrial membrane that plays crucial roles in the maintenance of crista junctions, inner membrane architecture, and formation of contact sites to the outer membrane	IMM	Cristae shape	[15,16,18,19,37]
INF2, C14orf151, C14orf173	Inverted formin-2 (HBEBP2-binding protein C)	Severs actin filaments and accelerates their polymerization and depolymerization	Cytosol	Mitochondrial constriction	[29,38]
LETM1	Mitochondrial proton/calcium exchanger protein (Leucine zipper-EF-hand-containing transmembrane protein 1)	Mitochondrial proton/calcium antiporter that mediates proton-dependent calcium efflux from mitochondria	IMM	Fission	[1,22,39]
MARCH5, RNF153	E3 ubiquitin-protein ligase MARCH5 (EC 2.3.2.27) (Membrane-associated RING finger protein 5) (Membrane-associated RING-CH protein V) (MARCH-V) (Mitochondrial ubiquitin ligase) (MITOL) (RING finger protein 153) (RING-type E3 ubiquitin transferase MARCH5)	Membrane-bound E3 ligase for mitochondrial morphology control	OMM	Fission	[1,19]
MAVS, IPS1, KIAA1271, VISA	Mitochondrial antiviral-signaling protein (MAVS) (CARD adapter inducing interferon beta) (Cardif) (Interferon beta promoter stimulator protein 1) (IPS-1) (Putative NF-kappa-B-activating protein 031N) (Virus-induced-signaling adapter) (VISA)	Required for innate immune response against viruses	OMM	Fusion	[22,40]
MCL1, BCL2L3	Induced myeloid leukemia cell differentiation protein Mcl-1 (Bcl-2-like protein 3) (Bcl2-L-3) (Bcl-2-related protein EAT/mcl1) (mcl1/EAT)	Regulation of apoptosis	IMM and OMM	Cristae shape (IMM isoform)	[18,29,41,42]
MFF, C2orf33, AD030, AD033, GL004	Mitochondrial Fission Factor	Mitochondrial and peroxisome division	OMM	Fission	[3,19]
MFN1	Mitofusin-1 (EC 3.6.5.-) (Fzo homolog) (Transmembrane GTPase MFN1)	Mitochondrial fusion	OMM	Fusion	[18,22,43]
MFN2, CPRP1, KIAA0214	Mitofusin-2 (EC 3.6.5.-) (Transmembrane GTPase MFN2)	Regulates mitochondrial clustering and fusion	OMM	Fusion	[18,22,43]
MIC13, C19orf70, QIL1	MICOS Complex subunit MIC13 (Protein P117)	Component of the MICOS Complex, a large protein Complex of the inner mitochondrial membrane that plays crucial roles in the maintenance of crista junctions, inner membrane architecture, and formation of contact sites to the outer membrane	IMM	Cristae shape	[15,18]
MIEF1, MID51, SMCR7L	Mid51/Mief, mitochondrial dynamics proteins of 51	Component of the MICOS Complex, a large protein Complex of the inner mitochondrial membrane that plays crucial roles in the maintenance of crista junctions, inner membrane architecture, and formation of contact sites to the outer membrane	OMM	Fission	[3,19]
MIEF2, MID49, SMCR7	Mitochondrial dynamics protein MID49 (Mitochondrial dynamics protein of 49 kDa) (Mitochondrial elongation factor 2) (Smith-Magenis syndrome chromosomal region candidate gene 7 protein)	Component of the MICOS Complex, a large protein Complex of the inner mitochondrial membrane that plays crucial roles in the maintenance of crista junctions, inner membrane architecture, and formation of contact sites to the outer membrane	OMM	Fission	[3,19]
MIGA1, FAM73A	Mitoguardin 1 (Protein FAM73A)	Regulator of mitochondrial fusion	OMM	Fusion	[4,44]
MIGA2, C9orf54, FAM73B, PSEC0112	Mitoguardin 2 (Protein FAM73B)	Regulator of mitochondrial fusion	OMM	Fusion	[4,44]
MINOS1, C1orf151, MIC10	MICOS Complex subunit MIC10 (Inner mitochondrial membrane organizing system protein 1)	Maintenance of cristae junctions, inner membrane architecture, and formation of contact sites to the outer membrane	IMM	Cristae shape	[15,18]
MTFP1, MTP18, HSPC242, My022	Mitochondrial fission process protein 1(Mitochondrial 18 kDa protein) (MTP18)	Involved in the mitochondrial division probably by regulating membrane fission	IMM	Fission	[1,22,45]
MUL1, C1orf166, GIDE, MAPL, MULAN, RNF218	Mitochondrial ubiquitin ligase activator of NFKB 1 (EC 2.3.2.27) (E3 SUMO-protein ligase MUL1) (E3 ubiquitin-protein ligase MUL1) (Growth inhibition and death E3 ligase) (Mitochondrial-anchored protein ligase) (MAPL) (Putative NF-kappa-B-activating protein 266) (RING finger protein 218) (RING-type E3 ubiquitin transferase NFKB 1)	Ubiquitin ligase activity	OMM	Fusion	[19,29]
NFE2L2, NRF2	Nuclear factor erythroid 2-related factor 2 (NF-E2-related factor 2) (NFE2-related factor 2) (HEBP1) (Nuclear factor, erythroid derived 2, like 2)	Transcription activator that binds to antioxidant response (ARE) elements in the promoter regions of target genes	Cytosol	Fusion	[29,46,47]
NRF1	Nuclear respiratory factor 1 (NRF-1) (Alpha palindromic-binding protein) (Alpha-pal)	Transcription factor implicated in the control of nuclear genes required for respiration, heme biosynthesis, and mitochondrial DNA transcription and replication	Cytosol	Fission	[29,46,48,49]
OMA1, MPRP1	Metalloendopeptidase OMA1, mitochondrial (EC 3.4.24.-) (Metalloprotease-related protein 1) (MPRP-1) (Overlapping with the m-AAA protease 1 homolog)	Metalloprotease that is part of the quality control system in the inner membrane of mitochondria	IMM	Fusion	[1,19]
OPA1, KIAA0567	Dynamin-like 120 kDa protein, mitochondrial (EC 3.6.5.5) (Optic atrophy protein 1) (Cleaved into: Dynamin-like 120 kDa protein, form S1)	Opa1 mediates dynamics changes in cristae morphology that correlate with the metabolic state of the organelle	IMM	Cristae shape, fusion	[1,3,15,18,22,50]
PARL, PSARL, PRO2207	Presenilins-associated rhomboid-like protein, mitochondrial (EC 3.4.21.105) (Mitochondrial intramembrane cleaving protease PARL) (Cleaved into: P-beta (Pbeta))	Required for the control of apoptosis	IMM	Mitochondrial morphology	[1,51]
PGAM5	Serine/threonine-protein phosphatase PGAM5, mitochondrial (EC 3.1.3.16) (Bcl-XL-binding protein v68) (Phosphoglycerate mutase family member 5)	Displays phosphatase activity for serine/threonine residues, as well as dephosphorylates and activates MAP3K5 kinase	OMM	Fission	[1,29,52]
PHB	Prohibitin	Prohibitin inhibits DNA synthesis; it has a role in regulating proliferation	IMM	Cristae shape	[1,15,18,31,53]
PHB2, BAP, REA	Prohibitin-2 (B-cell receptor-associated protein BAP37) (D-prohibitin) (Repressor of estrogen receptor activity)	Acts as a mediator of transcriptional repression by nuclear hormone receptors via the recruitment of histone deacetylases (by similarity); functions as an estrogen receptor (ER)-selective coregulator that potentiates the inhibitory activities of antiestrogens and represses the activity of estrogens	IMM	Cristae shape	[1,15,18]
PINK1	Serine/threonine-protein kinase PINK1, mitochondrial (EC 2.7.11.1) (BRPK) (PTEN-induced putative kinase protein 1)	Protects against mitochondrial dysfunction during cellular stress by phosphorylating mitochondrial proteins	OMM	Fission	[1,54]
PLD6	Mitochondrial cardiolipin hydrolase (EC 3.1.-.-) (Choline phosphatase 6) (Mitochondrial phospholipase) (MitoPLD) (Phosphatidylcholine-hydrolyzing phospholipase D6) (Phospholipase D6) (PLD 6) (Protein zucchini homolog)	Proposed to act as a cardiolipin hydrolase to generate phosphatidic acid at the mitochondrial surface	OMM	Fusion	[4,19,22,45]
PPARGC1A, LEM6, PGC1, PGC1A, PPARGC1	Peroxisome proliferator-activated receptor gamma coactivator 1-alpha (PGC-1-alpha) (PPAR-gamma coactivator 1-alpha) (PPARGC-1-alpha) (Ligand effect modulator 6)	Transcriptional coactivator for steroid receptors and nuclear receptors	Cytoplasm and nucleus	Fusion	[29,49,55]
PPARGC1B, PERC, PGC1, PGC1B, PPARGC1	Peroxisome proliferator-activated receptor gamma coactivator 1-beta (PGC-1-beta) (PPAR-gamma coactivator 1-beta) (PPARGC-1-beta) (PGC-1-related estrogen receptor alpha coactivator)	Plays the role of stimulator of transcription factors and nuclear receptors activities	Nucleus	Fusion	[29,55,56]
PRELID1, PRELI, CGI-106, SBBI12	PRELI domain-containing protein 1, mitochondrial (25 kDa protein of relevant evolutionary and lymphoid interest) (Px19-like protein)	Involved in the modulation of the mitochondrial apoptotic pathway by ensuring the accumulation of cardiolipin (CL) in mitochondrial membranes	Intermembrane space	Fission	[57,58]
PRKN, PARK2	E3 ubiquitin-protein ligase parkin (Parkin) (EC 2.3.2.-) (Parkin RBR E3 ubiquitin-protein ligase) (Parkinson juvenile disease protein 2) (Parkinson disease protein 2)	Functions within a multiprotein E3 ubiquitin ligase Complex, catalyzing the covalent attachment of ubiquitin moieties onto substrate proteins	Cytosol and mitochondria	Fission	[1,54,59,60]
ROMO1, C20orf52	Reactive oxygen species modulator 1(ROS modulator 1) (Epididymis tissue protein Li 175) (Glyrichin) (Mitochondrial targeting GxxxG motif protein) (MTGM) (Protein MGR2 homolog)	Induces the production of reactive oxygen species (ROS), which are necessary for cell proliferation	IMM	Fusion	[29,61]
SAMM50, SAM50, CGI-51, TRG3	Sorting and assembly machinery component 50 homolog (Transformation-related gene 3 protein) (TRG-3)	Plays a crucial role in the maintenance of the structure of mitochondrial cristae and the proper assembly of the mitochondrial respiratory chain Complexes	OMM	Cristae shape	[18]
SH3GLB1, KIAA0491, CGI-61	Endophilin-B1 (Bax-interacting factor 1) (Bif-1) (SH3 domain-containing GRB2-like protein B1)	Outer mitochondrial dynamics	OMM	OMM permeability	[1,22,62]
SH3GLB2, KIAA1848, PP578	Endophilin-B2 (SH3 domain-containing GRB2-like protein B2)	Mitophagy	Cytosol	Fission	[63]
SLC25A10, DIC	Mitochondrial dicarboxylate carrier (Solute carrier family 25 member 10)	Involved in the translocation of malonate, malate, and succinate in exchange for phosphate, sulfate, sulfite, or thiosulfate across the inner mitochondrial membrane	IMM	Cristae shape	[18,50]
SLC25A11, SLC20A4	Mitochondrial 2-oxoglutarate/malate carrier protein (OGCP) (Solute carrier family 25 member 11)	Catalyzes the transport of 2-oxoglutarate across the inner mitochondrial membrane in an electroneutral exchange for malate or other dicarboxylic acids, and plays an important role in several metabolic processes, including the malate-aspartate shuttle, the oxoglutarate/isocitrate shuttle, in gluconeogenesis from lactate, and in nitrogen metabolism	IMM	Cristae shape	[18,50]
SLC25A12, ARALAR1	Calcium-binding mitochondrial carrier protein Aralar1 (Mitochondrial aspartate glutamate carrier 1) (Solute carrier family 25 member 12)	Catalyzes the calcium-dependent exchange of cytoplasmic glutamate with mitochondrial aspartate across the inner mitochondrial membrane; may have a function in the urea cycle	IMM	Cristae shape	[18,50]
SLC25A13, ARALAR2	Calcium-binding mitochondrial carrier protein Aralar2 (Citrin) (Mitochondrial aspartate glutamate carrier 2) (Solute carrier family 25 member 13)	Catalyzes the calcium-dependent exchange of cytoplasmic glutamate with mitochondrial aspartate across the inner mitochondrial membrane; may have a function in the urea cycle	IMM	Cristae shape	[18,50]
SLC25A38	Solute carrier family 25 member 38, Appoptosin	Mitochondrial import machinery	IMM	Fusion	[29,64]
SMAD2, MADH2, MADR2	Mothers against decapentaplegic homolog 2 (MAD homolog 2) (Mothers against DPP homolog 2) (JV18-1) (Mad-related protein 2) (hMAD-2) (SMAD family member 2) (SMAD 2) (Smad2) (hSMAD2)	Receptor-regulated SMAD (R-SMAD) that is an intracellular signal transducer and transcriptional modulator activated by TGF-beta (transforming growth factor) and activin type 1 receptor kinases	Cytosol	Fusion	[29,65]
SPG7, CAR, CMAR, PGN	Paraplegin (EC 3.4.24.-) (Cell matrix adhesion regulator) (Spastic paraplegia 7 protein)	ATP-dependent zinc metalloprotease	IMM	Cristae shape	[18]
SPG7, CAR, CMAR, PGN	Paraplegin (EC 3.4.24.-) (Cell matrix adhesion regulator) (Spastic paraplegia 7 protein)	ATP-dependent zinc metalloprotease	IMM	Fusion	[1,22,51]
STOML2, SLP2, HSPC108	Stomatin-like protein 2, mitochondrial(SLP-2) (EPB72-like protein 2) (Paraprotein target 7) (Paratarg-7)	Mitochondrial protein that probably regulates the biogenesis and the activity of mitochondria; stimulates cardiolipin biosynthesis, binds cardiolipin-enriched membranes where it recruits and stabilizes some proteins including prohibitin, and may therefore act in the organization of functional microdomains in mitochondrial membranes	IMM	Cristae shape/Stabilize IM structure	[15,18]
SYNJ2, KIAA0348	Synaptojanin-2(EC 3.1.3.36) (Synaptic inositol 1,4,5-trisphosphate 5-phosphatase 2)	Membrane trafficking and signaling transduction	Cytosol	Mitochondrial aggregation	[66]
TAZ, EFE2, G4.5	Tafazzin (Protein G4.5)	Some isoforms may be involved in cardiolipin (CL) metabolism	OMM	Cristae shape	[15,16,67,68]
TFAM, TCF6, TCF6L2	Transcription factor A, mitochondrial (mtTFA) (Mitochondrial transcription factor 1) (MtTF1) (Transcription factor 6) (TCF-6) (Transcription factor 6-like 2)	Binds to the mitochondrial light strand promoter and functions in mitochondrial transcription regulation	Matrix	Mitochondrial biogenesis	[29,46]
TRAK1, KIAA1042, OIP106	Trafficking kinesin-binding protein 1 (106 kDa O-GlcNAc transferase-interacting protein)	Organelle trafficking	OMM and cytosol	Fusion	[1,4,19]
TRAK2, ALS2CR3, KIAA0549	Trafficking kinesin-binding protein 2 (Amyotrophic lateral sclerosis 2 chromosomal region candidate gene 3 protein)	Organelle trafficking	OMM and cytosol	Fusion	[1,4,19]
UQCC3, C11orf83, UNQ655/PRO1286	Ubiquinol-cytochrome-c reductase Complex assembly factor 3	Required for the assembly of the ubiquinol-cytochrome c reductase Complex (mitochondrial respiratory chain Complex III or cytochrome b-c1 Complex), mediating cytochrome b recruitment and probably stabilization within the Complex	IMM	Cristae shape	[18,69]
VAT1	Synaptic vesicle membrane protein VAT-1 homolog (EC 1.-.-.-) (Mitofusin-binding protein) (Protein MIB)	Negatively regulates mitochondrial fusion	OMM	Fusion	[1,19,22,70]
YME1L1, FTSH1, YME1L, UNQ1868/PRO4304	ATP-dependent zinc metalloprotease YME1L1 (EC 3.4.24.-) (ATP-dependent metalloprotease FtsH1) (Meg-4) (Presenilin-associated metalloprotease) (PAMP) (YME1-like protein 1)	Putative ATP-dependent protease; plays a role in mitochondrial organization and mitochondrial protein metabolism, including the degradation of PRELID1 and OPA1	IMM	Cristae shape	[19,58]

**Table 2:** List of mitochondrial-shaping proteins found to be modified in Parkinson’s disease.

Gene Symbol (bioDBnet)	Name	Mito_Shaping
APOO, FAM121B, MIC23, MIC26, My025, UNQ1866/PRO4302	MICOS Complex subunit MIC26 (Apolipoprotein O) (MICOS Complex subunit MIC23) (Protein FAM121B)	Cristae shape
ATP5A1, ATP5A, ATP5AL2, ATPM	ATP synthase subunit alpha, mitochondrial	Cristae shape
ATP5B, ATPMB, ATPSB	ATP synthase subunit beta, mitochondrial	Cristae shape
ATP5H	ATP synthase subunit d	Cristae shape
ATP5I	ATP synthase subunit e, mitochondrial	Cristae shape
SAMM50, SAM50, CGI-51, TRG3	Sorting and assembly machinery component 50 homolog (Transformation-related gene 3 protein) (TRG-3)	Cristae shape
COX4I1, COX4	Cytochrome c oxidase subunit 4 isoform 1, mitochondrial (Cytochrome c oxidase polypeptide IV) (Cytochrome c oxidase subunit IV isoform 1) (COX IV-1)	Cristae shape
COX5A	Cytochrome c oxidase subunit 5A, mitochondrial (Cytochrome c oxidase polypeptide Va)	Cristae shape
COX6C	Cytochrome c oxidase subunit 6C (Cytochrome c oxidase polypeptide VIc)	Cristae shape
COX7C	Cytochrome c oxidase subunit 7C, mitochondrial (Cytochrome c oxidase polypeptide VIIc)	Cristae shape
MFN2, CPRP1, KIAA0214	Mitofusin-2 (EC 3.6.5.-) (Transmembrane GTPase MFN2)	Fusion
DNM1L	Dynamin-like protein	Fission
IMMT	Mitofilin	Cristae shape
MT-CO1	Cytochrome c oxidase polypeptide I	Cristae shape
PHB	Prohibitin	Cristae shape
PHB2	Prohibitin-2	Cristae shape
SH3GLB2	SH3-containing protein SH3GLB2	Fission
SLC25A13	Calcium-binding mitochondrial carrier protein Aralar2	Cristae shape
UQCRB, UQBP	Cytochrome b-c1 Complex subunit 7 (Complex III subunit 7) (Complex III subunit VII) (QP-C) (Ubiquinol-cytochrome c reductase Complex 14 kDa protein)	Cristae shape
UQCRFS1	Ubiquinol cytochrome c reductase iron–sulfur subunit	Cristae shape
UQCRH	Cytochrome b-c1 Complex subunit 6, mitochondrial	Cristae shape
VAT1	Synaptic vesicle membrane protein VAT-1 homolog	Fusion

**Table 3:** List of binding partners of the mitochondrial-shaping proteins altered in Parkinson’s disease (Table 2) that are found in the list of proteins modified in the context of Parkinson’s disease (Supplementary Table S1).

Gene Names	Protein Names
ACAD9	Acyl-CoA dehydrogenase family member 9, mitochondrial (ACAD-9) (EC 1.3.99.-)
ACP2	Lysosomal acid phosphatase (LAP) (EC 3.1.3.2)
ACTB	Actin, cytoplasmic 1 (Beta-actin) (Cleaved into: Actin, cytoplasmic 1, *N*-terminally processed)
ACTBL2	Beta-actin-like protein 2 (Kappa-actin)
ACTN1	Alpha-actinin-1 (Alpha-actinin cytoskeletal isoform) (F-actin cross-linking protein) (Non-muscle alpha-actinin-1)
ALB, GIG20, GIG42, PRO0903, PRO1708, PRO2044, PRO2619, PRO2675, UNQ696/PRO1341	Serum albumin
ALDH1B1, ALDH5, ALDHX	Aldehyde dehydrogenase X, mitochondrial (EC 1.2.1.3) (Aldehyde dehydrogenase 5) (Aldehyde dehydrogenase family 1 member B1)
ALDOA, ALDA	Fructose-bisphosphate aldolase A (EC 4.1.2.13) (Lung cancer antigen NY-LU-1) (Muscle-type aldolase)
ANXA2, ANX2, ANX2L4, CAL1H LPC2D	Annexin A2 (Annexin II) (Annexin-2) (Calpactin I heavy chain) (Calpactin-1 heavy chain) (Chromobindin-8) (Lipocortin II) (Placental anticoagulant protein IV) (PAP-IV) (Protein I) (p36)
APOA1	Apolipoprotein A-I (Apo-AI) (ApoA-I) (Apolipoprotein A1) (Cleaved into: Proapolipoprotein A-I (Proapo A-I); Truncated apolipoprotein A-I (Apolipoprotein A-I(1-242)))
ATP5A1, ATP5A, ATP5AL2, ATPM	ATP synthase subunit alpha, mitochondrial
ATP5B, ATPMB, ATPSB,	ATP synthase subunit beta, mitochondrial (EC 3.6.3.14)
ATP5H, My032	ATP synthase subunit d, mitochondrial (ATPase subunit d)
ATP5I, ATP5K	ATP synthase subunit e, mitochondrial (ATPase subunit e) (Cleaved into: ATP synthase subunit e, mitochondrial, *N*-terminally processed)
BCAP31, BAP31, DXS1357E	B-cell receptor-associated protein 31 (BCR-associated protein 31) (Bap31) (6C6-AG tumor-associated antigen) (Protein CDM) (p28)
C1QBP, GC1QBP, HABP1, SF2P32	Complement component 1 Q subcomponent-binding protein, mitochondrial (ASF/SF2-associated protein p32) (Glycoprotein gC1qBP) (C1qBP) (Hyaluronan-binding protein 1) (Mitochondrial matrix protein p32) (gC1q-R protein) (p33)
CALR, CRTC	Calreticulin (CRP55) (Calregulin) (Endoplasmic reticulum resident protein 60) (ERp60) (HACBP) (grp60)
CCT5, CCTE, KIAA0098	T-complex protein 1 subunit epsilon (TCP-1-epsilon) (CCT-epsilon)
COX4I1, COX4	Cytochrome c oxidase subunit 4 isoform 1, mitochondrial (Cytochrome c oxidase polypeptide IV) (Cytochrome c oxidase subunit IV isoform 1) (COX IV-1)
COX5A	Cytochrome c oxidase subunit 5A, mitochondrial (Cytochrome c oxidase polypeptide Va)
COX6C	Cytochrome c oxidase subunit 6C (Cytochrome c oxidase polypeptide VIc)
DDAH1, DDAH	N(G),N(G)-dimethylarginine dimethylaminohydrolase 1 (DDAH-1) (Dimethylarginine dimethylaminohydrolase 1) (EC 3.5.3.18) (DDAHI) (Dimethylargininase-1)
DDOST, KIAA0115, OST48 OK/SW-cl.45	Dolichyl-diphosphooligosaccharide—protein glycosyltransferase 48 kDa subunit (DDOST 48 kDa subunit) (Oligosaccharyl transferase 48 kDa subunit)
DNM1L, DLP1, DRP1	Dynamin-1-like protein (EC 3.6.5.5) (Dnm1p/Vps1p-like protein) (DVLP) (Dynamin family member proline-rich carboxyl-terminal domain less) (Dymple) (Dynamin-like protein) (Dynamin-like protein 4) (Dynamin-like protein IV) (HdynIV) (Dynamin-related protein 1)
DYNC1H1, DHC1, DNCH1, DNCL, DNECL, DYHC, KIAA0325	Cytoplasmic dynein 1 heavy chain 1 (Cytoplasmic dynein heavy chain 1) (Dynein heavy chain, cytosolic)
EEF1A1, EEF1A, EF1A, LENG7	Elongation factor 1-alpha 1 (EF-1-alpha-1) (Elongation factor Tu) (EF-Tu) (Eukaryotic elongation factor 1 A-1) (eEF1A-1) (Leukocyte receptor cluster member 7)
EEF1B2, EEF1B, EF1B	Elongation factor 1-beta (EF-1-beta)
EIF5A	Eukaryotic translation initiation factor 5A-1 (eIF-5A-1) (eIF-5A1) (Eukaryotic initiation factor 5A isoform 1) (eIF-5A) (Rev-binding factor) (eIF-4D)
FKBP4, FKBP52	Peptidyl-prolyl cis-trans isomerase FKBP4 (PPIase FKBP4) (EC 5.2.1.8) (51 kDa FK506-binding protein) (FKBP51) (52 kDa FK506-binding protein) (52 kDa FKBP) (FKBP-52) (59 kDa immunophilin) (p59) (FK506-binding protein 4) (FKBP-4) (FKBP59) (HSP-binding immunophilin) (HBI) (Immunophilin FKBP52) (Rotamase) (Cleaved into: Peptidyl-prolyl cis-trans isomerase FKBP4, *N*-terminally processed)
FLNC, ABPL, FLN2	Filamin-C (FLN-C) (FLNc) (ABP-280-like protein) (ABP-L) (Actin-binding-like protein) (Filamin-2) (Gamma-filamin)
FLOT1	Flotillin-1
FUBP1	Far upstream element-binding protein 1 (FBP) (FUSE-binding protein 1) (DNA helicase V) (hDH V)
GARS	Glycine-tRNA ligase (EC 3.6.1.17) (EC 6.1.1.14) (Diadenosine tetraphosphate synthetase) (AP-4-A synthetase) (Glycyl-tRNA synthetase) (GlyRS)
GSTK1, HDCMD47P	Glutathione S-transferase kappa 1 (EC 2.5.1.18) (GST 13-13) (GST class-kappa) (GSTK1-1) (hGSTK1) (Glutathione S-transferase subunit 13)
GSTO1, GSTTLP28	Glutathione S-transferase omega-1 (GSTO-1) (EC 2.5.1.18) (Glutathione S-transferase omega 1-1) (GSTO 1-1) (Glutathione-dependent dehydroascorbate reductase) (EC 1.8.5.1) (Monomethylarsonic acid reductase) (MMA(V) reductase) (EC 1.20.4.2) (S-(Phenacyl)glutathione reductase) (SPG-R)
HSP90AB1, HSP90B HSPC2, HSPCB	Heat shock protein HSP 90-beta (HSP 90) (Heat shock 84 kDa) (HSP 84) (HSP84)
HSPA1L	Heat shock 70 kDa protein 1-like (Heat shock 70 kDa protein 1L) (Heat shock 70 kDa protein 1-Hom) (HSP70-Hom)
HSPA5, GRP78	78 kDa glucose-regulated protein (GRP-78) (Endoplasmic reticulum lumenal Ca(2+)-binding protein grp78) (Heat shock 70 kDa protein 5) (Immunoglobulin heavy chain-binding protein) (BiP)
HSPA8, HSC70, HSP73, HSPA10	Heat shock cognate 71 kDa protein (Heat shock 70 kDa protein 8) (Lipopolysaccharide-associated protein 1) (LAP-1) (LPS-associated protein 1)
HSPA9, GRP75, HSPA9B, mt-HSP70	Stress-70 protein, mitochondrial (75 kDa glucose-regulated protein) (GRP-75) (Heat shock 70 kDa protein 9) (Mortalin) (MOT) (Peptide-binding protein 74) (PBP74)
HSPB1, HSP27, HSP28	Heat shock protein beta-1 (HspB1) (28 kDa heat shock protein) (Estrogen-regulated 24 kDa protein) (Heat shock 27 kDa protein) (HSP 27) (Stress-responsive protein 27) (SRP27)
HSPD1, HSP60	60 kDa heat shock protein, mitochondrial (EC 3.6.4.9) (60 kDa chaperonin) (Chaperonin 60) (CPN60) (Heat shock protein 60) (HSP-60) (Hsp60) (HuCHA60) (Mitochondrial matrix protein P1) (P60 lymphocyte protein)
ILVBL, AHAS	Acetolactate synthase-like protein (EC 2.2.1.-) (IlvB-like protein)
IMMT, HMP, MIC60, MINOS2, PIG4, PIG52	MICOS Complex subunit MIC60 (Cell proliferation-inducing gene 4/52 protein) (Inner mitochondrial membrane protein) (Mitofilin) (p87/89)
LDHB	L-lactate dehydrogenase B chain (LDH-B) (EC 1.1.1.27) (LDH heart subunit) (LDH-H) (Renal carcinoma antigen NY-REN-46)
LGALS1	Galectin-1 (Gal-1) (14 kDa laminin-binding protein) (HLBP14) (14 kDa lectin) (Beta-galactoside-binding lectin L-14-I) (Galaptin) (HBL) (HPL) (Lactose-binding lectin 1) (Lectin galactoside-binding soluble 1) (Putative MAPK-activating protein PM12) (S-Lac lectin 1)
LMNA, LMN1	Prelamin-A/C (Cleaved into: Lamin-A/C (70 kDa lamin) (Renal carcinoma antigen NY-REN-32))
MDH2	Malate dehydrogenase, mitochondrial (EC 1.1.1.37)
MFN2, CPRP1, KIAA0214	Mitofusin-2 (EC 3.6.5.-) (Transmembrane GTPase MFN2)
MYL6	Myosin light polypeptide 6 (17 kDa myosin light chain) (LC17) (Myosin light chain 3) (MLC-3) (Myosin light chain alkali 3) (Myosin light chain A3) (Smooth muscle and non-muscle myosin light chain alkali 6)
NDUFA10	NADH dehydrogenase (ubiquinone) 1 alpha subcomplex subunit 10, mitochondrial (Complex I-42kD) (CI-42kD) (NADH-ubiquinone oxidoreductase 42 kDa subunit)
NDUFA11	NADH dehydrogenase [ubiquinone] 1 alpha subcomplex subunit 11 (Complex I-B14.7) (CI-B14.7) (NADH-ubiquinone oxidoreductase subunit B14.7)
NDUFA4	Cytochrome c oxidase subunit NDUFA4 (Complex I-MLRQ) (CI-MLRQ) (NADH-ubiquinone oxidoreductase MLRQ subunit)
NDUFS1	NADH-ubiquinone oxidoreductase 75 kDa subunit, mitochondrial (EC 1.6.5.3) (EC 1.6.99.3) (Complex I-75kD) (CI-75kD)
NDUFS3	NADH dehydrogenase (ubiquinone) iron-sulfur protein 3, mitochondrial (EC 1.6.5.3) (EC 1.6.99.3) (Complex I-30kD) (CI-30kD) (NADH-ubiquinone oxidoreductase 30 kDa subunit)
NEDD8	NEDD8 (Neddylin) (Neural precursor cell expressed developmentally downregulated protein 8) (NEDD-8) (Ubiquitin-like protein Nedd8)
NPM1, NPM	Nucleophosmin (NPM) (Nucleolar phosphoprotein B23) (Nucleolar protein NO38) (Numatrin)
OAT	Ornithine aminotransferase, mitochondrial (EC 2.6.1.13) (Ornithine delta-aminotransferase) (Ornithine—oxo-acid aminotransferase) (Cleaved into: Ornithine aminotransferase, hepatic form; Ornithine aminotransferase, renal form)
OGDH	2-oxoglutarate dehydrogenase, mitochondrial (EC 1.2.4.2) (2-oxoglutarate dehydrogenase complex component E1) (OGDC-E1) (Alpha-ketoglutarate dehydrogenase)
OTUB1, OTB1, OTU1, HSPC263	Ubiquitin thioesterase OTUB1 (EC 3.4.19.12) (Deubiquitinating enzyme OTUB1) (OTU domain-containing ubiquitin aldehyde-binding protein 1) (Otubain-1) (hOTU1) (Ubiquitin-specific-processing protease OTUB1)
PDIA3, ERP57, ERP60, GRP58	Protein disulfide-isomerase A3 (EC 5.3.4.1) (58 kDa glucose-regulated protein) (58 kDa microsomal protein) (p58) (Disulfide isomerase ER-60) (Endoplasmic reticulum resident protein 57) (ER protein 57) (ERp57) (Endoplasmic reticulum resident protein 60) (ER protein 60) (ERp60)
PGK1, PGKA, MIG10, OK/SW-cl.110	Phosphoglycerate kinase 1 (EC 2.7.2.3) (Cell migration-inducing gene 10 protein) (Primer recognition protein 2) (PRP 2)
PHB	Prohibitin
PHB2, BAP, REA	Prohibitin-2 (B-cell receptor-associated protein BAP37) (D-prohibitin) (Repressor of estrogen receptor activity)
PIN1	Peptidyl-prolyl cis-trans isomerase NIMA-interacting 1 (EC 5.2.1.8) (Peptidyl-prolyl cis-trans isomerase Pin1) (PPIase Pin1) (Rotamase Pin1)
PPIA, CYPA	Peptidyl-prolyl cis-trans isomerase A (PPIase A) (EC 5.2.1.8) (Cyclophilin A) (Cyclosporin A-binding protein) (Rotamase A) (Cleaved into: Peptidyl-prolyl cis-trans isomerase A, *N*-terminally processed)
PRDX4	Peroxiredoxin-4 (EC 1.11.1.15) (Antioxidant enzyme AOE372) (AOE37-2) (Peroxiredoxin IV) (Prx-IV) (Thioredoxin peroxidase AO372) (Thioredoxin-dependent peroxide reductase A0372)
PSMA3, HC8, PSC8	Proteasome subunit alpha type-3 (EC 3.4.25.1) (Macropain subunit C8) (Multi-catalytic endopeptidase complex subunit C8) (Proteasome component C8)
PTPN5	Tyrosine-protein phosphatase non-receptor type 5 (EC 3.1.3.48) (Neural-specific protein-tyrosine phosphatase) (Striatum-enriched protein-tyrosine phosphatase) (STEP)
RAB14	Ras-related protein Rab-14
RAB2A, RAB2	Ras-related protein Rab-2A
RAC1, TC25, MIG5	Ras-related C3 botulinum toxin substrate 1 (Cell migration-inducing gene 5 protein) (Ras-like protein TC25) (p21-Rac1)
RAPGEF2, KIAA0313, NRAPGEP, PDZGEF1	Rap guanine nucleotide exchange factor 2 (Cyclic nucleotide ras GEF) (CNrasGEF) (Neural RAP guanine nucleotide exchange protein) (nRap GEP) (PDZ domain-containing guanine nucleotide exchange factor 1) (PDZ-GEF1) (RA-GEF-1) (Ras/Rap1-associating GEF-1)
RHOA, ARH12, ARHA, RHO12	Transforming protein RhoA (Rho cDNA clone 12) (h12)
RNH1, PRI, RNH	Ribonuclease inhibitor (Placental ribonuclease inhibitor) (Placental RNase inhibitor) (Ribonuclease/angiogenin inhibitor 1) (RAI)
RPN1	Dolichyl-diphosphooligosaccharide—protein glycosyltransferase subunit 1 (Dolichyl-diphosphooligosaccharide—protein glycosyltransferase 67 kDa subunit) (Ribophorin I) (RPN-I) (Ribophorin-1)
RPN2	Dolichyl-diphosphooligosaccharide—protein glycosyltransferase subunit 2 (Dolichyl-diphosphooligosaccharide—protein glycosyltransferase 63 kDa subunit) (RIBIIR) (Ribophorin II) (RPN-II) (Ribophorin-2)
RPS15A, OK/SW-cl.82	40S ribosomal protein S15a (Small ribosomal subunit protein uS8)
RPS3, OK/SW-cl.26	40S ribosomal protein S3 (EC 4.2.99.18) (Small ribosomal subunit protein uS3)
S100A10, ANX2LG CAL1L CLP11	Protein S100-A10 (Calpactin I light chain) (Calpactin-1 light chain) (Cellular ligand of annexin II) (S100 calcium-binding protein A10) (p10 protein) (p11)
SAMM50, SAM50 CGI-51 TRG3	Sorting and assembly machinery component 50 homolog (Transformation-related gene 3 protein) (TRG-3)
SELENBP1, SBP	Selenium-binding protein 1 (56 kDa selenium-binding protein) (SBP56) (SP56)
SFXN1	Sideroflexin-1 (Tricarboxylate carrier protein) (TCC)
SH3GLB2, KIAA1848, PP578	Endophilin-B2 (SH3 domain-containing GRB2-like protein B2)
SIRT2, SIR2L, SIR2L2	NAD-dependent protein deacetylase sirtuin-2 (EC 3.5.1.-) (Regulatory protein SIR2 homolog 2) (SIR2-like protein 2)
SLC25A13, ARALAR2	Calcium-binding mitochondrial carrier protein Aralar2 (Citrin) (Mitochondrial aspartate glutamate carrier 2) (Solute carrier family 25 member 13)
SLC25A18, GC2	Mitochondrial glutamate carrier 2 (GC-2) (Glutamate/H(+) symporter 2) (Solute carrier family 25 member 18)
SLC25A5, ANT2	ADP/ATP translocase 2 (ADP, ATP carrier protein 2) (ADP, ATP carrier protein, fibroblast isoform) (Adenine nucleotide translocator 2) (ANT 2) (Solute carrier family 25 member 5) (Cleaved into: ADP/ATP translocase 2, *N*-terminally processed)
SLC9A3R1, NHERF, NHERF1	Na(+)/H(+) exchange regulatory cofactor NHE-RF1 (NHERF-1) (Ezrin-radixin-moesin-binding phosphoprotein 50) (EBP50) (Regulatory cofactor of Na(+)/H(+) exchanger) (Sodium-hydrogen exchanger regulatory factor 1) (Solute carrier family 9 isoform A3 regulatory factor 1)
SSBP1, SSBP	Single-stranded DNA-binding protein, mitochondrial (Mt-SSB) (MtSSB) (PWP1-interacting protein 17)
TPD52	Tumor protein D52 (Protein N8)
TPM1, C15orf13, TMSA	Tropomyosin alpha-1 chain (Alpha-tropomyosin) (Tropomyosin-1)
TPM2, TMSB	Tropomyosin beta chain (Beta-tropomyosin) (Tropomyosin-2)
TPM3	Tropomyosin alpha-3 chain (Gamma-tropomyosin) (Tropomyosin-3) (Tropomyosin-5) (hTM5)
TPM4	Tropomyosin alpha-4 chain (TM30p1) (Tropomyosin-4)
TUBA1A, TUBA3	Tubulin alpha-1A chain (Alpha-tubulin 3) (Tubulin B-alpha-1) (Tubulin alpha-3 chain) (Cleaved into: Detyrosinated tubulin alpha-1A chain)
TUBB, TUBB5, OK/SW-cl.56	Tubulin beta chain (Tubulin beta-5 chain)
TUBB4B, TUBB2C	Tubulin beta-4B chain (Tubulin beta-2 chain) (Tubulin beta-2C chain)
TUFM	Elongation factor Tu, mitochondrial (EF-Tu) (P43)
UQCRB, UQBP	Cytochrome b-c1 complex subunit 7 (Complex III subunit 7) (Complex III subunit VII) (QP-C) (Ubiquinol-cytochrome c reductase complex 14 kDa protein)
UQCRFS1	Cytochrome b-c1 complex subunit Rieske, mitochondrial (EC 1.10.2.2) (Complex III subunit 5) (Cytochrome b-c1 complex subunit 5) (Rieske iron-sulfur protein) (RISP) (Ubiquinol-cytochrome c reductase iron-sulfur subunit) (Cleaved into: Cytochrome b-c1 complex subunit 11 (Complex III subunit IX) (Ubiquinol-cytochrome c reductase 8 kDa protein))
UQCRFS1P1, UQCRFSL1	Putative cytochrome b-c1 complex subunit Rieske-like protein 1 (Ubiquinol-cytochrome c reductase Rieske iron-sulfur subunit pseudogene 1)
UQCRH	Cytochrome b-c1 complex subunit 6, mitochondrial (Complex III subunit 6) (Complex III subunit VIII) (Cytochrome c1 non-heme 11 kDa protein) (Mitochondrial hinge protein) (Ubiquinol-cytochrome c reductase complex 11 kDa protein)
USMG5, DAPIT, HCVFTP2, PD04912	Upregulated during skeletal muscle growth protein 5 (Diabetes-associated protein in insulin-sensitive tissues) (HCV F-transactivated protein 2)
VCP	Transitional endoplasmic reticulum ATPase (TER ATPase) (EC 3.6.4.6) (15S Mg(2+)-ATPase p97 subunit) (Valosin-containing protein) (VCP)
VDAC1, VDAC	Voltage-dependent anion-selective channel protein 1 (VDAC-1) (hVDAC1) (Outer mitochondrial membrane protein porin 1) (Plasmalemmal porin) (Porin 31HL) (Porin 31HM)
VDAC2	Voltage-dependent anion-selective channel protein 2 (VDAC-2) (hVDAC2) (Outer mitochondrial membrane protein porin 2)
VDAC3	Voltage-dependent anion-selective channel protein 3 (VDAC-3) (hVDAC3) (Outer mitochondrial membrane protein porin 3)
VIM	Vimentin
YWHAE	14-3-3 protein epsilon (14-3-3E)
YWHAZ	14-3-3 protein zeta/delta (Factor activating exoenzyme S) (FAS) (Protein kinase C inhibitor protein 1) (KCIP-1)

**Table 4:** List of biological processes related to the mitochondrial-shaping proteins affected in Parkinson’s disease.

Upregulated	Downregulated
ATP biosynthetic process	ATP metabolic process
Positive regulation of nitric oxide biosynthetic process	Membrane raft assembly
Response to unfolded protein	Positive regulation of stress fiber assembly
Auditory receptor cell morphogenesis	Gluthatione derivative biosynthetic process
Binding of sperm to zona pellucida	Protein N-linked glycosylation via asparagine
Regulation of protein dephosphorylation	Glucocorticoid receptor signaling pathway
Mitochondrial electron transport ubiquinol to cytochrome c	ER-nucleus signaling pathway
Regulation of exit from mitosis	Substantia nigra development
NAD metabolic process	Positive regulation of NFkB signaling
Muscle filament sliding	Mitochondrial transmembrane transport
Regulation of complement activation	Mitochondrial electron transport cytochrome c to oxygen
Midbrain development and positive regulation of neutrophil chemotaxis	Respiratory electron transport chain
	Oxidative phosphorylation
	Mitochondrial respiratory chain Complex I assembly
	Mitochondrial electron transport NADH to ubiquinone
	Mitochondria respiratory chain complex assembly
	Mitochondrial ATP synthesis coupled to electron transport
	Cellular respiration and proton transport

## Supplementary Materials

The following are available online at www.mdpi.com/2076-3921/7/1/1/s1, Table S1: List of proteins found modified in different proteomics-based approaches in the context of Parkinson’s disease; Table S2: List of the binding partners of the “mitochondrial-shaping proteins found modified in Parkinson’s disease”; Table S3: List of the binding partners of the “mitochondrial-shaping proteins found modified in different proteomics-based approaches in the context of Parkinson’s disease according to the Human Integrated Protein-Protein Interaction reference” that are modified in Parkinson’s disease.

## Abbreviations

ABPL	actin-binding-like protein
ACAD9	acyl-CoA dehydrogenase family member 9
ACP2	acid phosphatase 2, lysosomal
ACTB	actin beta
ACTBL2	actin, beta like 2
ACTN1	actinin alpha 1
AD030	ORF name for Mitochondrial Fission Factor
AD033	ORF name for mitochondrial Fission Factor
ADP	adenosine diphosphate
AHAS	acetolactate synthase-like protein
ALB	albumin
ALDA	aldolase, fructose-bisphosphate A
ALDH1B1	aldehyde dehydrogenase 1 family member B1
ALDH5	Aldehyde dehydrogenase 5
ALDHX	Aldehyde dehydrogenase X
ALDOA	aldolase, fructose-bisphosphate A
ALS2CR3	Amyotrophic lateral sclerosis 2 chromosomal region candidate gene 3 protein
ANT2	Adenine nucleotide translocator 2
ANX2	annexin 2
ANX2L4	annexin A2
ANX2LG	annexin A2 ligand
ANXA2	annexin A2
APOA1	apolipoprotein A1
APOO	apolipoprotein O
APOOL	apolipoprotein O like
ARALAR1/AGC1	aspartate-glutamate carrier 1
ARALAR2/AGC2	aspartate-glutamate carrier 2
ARH12	Aplysia ras-related homolog 12
ARHA	Ras Homolog Gene Family, Member A
ATP	adenosine triphosphate
ATP5A	ATP synthase subunit alpha
ATP5A1	ATP synthase subunit alpha subunit 1
ATP5AL2	ATP synthase subunit alpha
ATP5B	ATP synthase subunit beta
ATP5C1	ATP synthase subunit gamma subunit 1
ATP5D	ATP synthase subunit delta
ATP5E	ATP synthase subunit epsilon
ATP5F1	ATP synthase F(0) complex subunit B1
ATP5G1	ATP synthase F(0) complex subunit C1
ATP5G2	ATP synthase F(0) complex subunit C2
ATP5G3	ATP synthase F(0) complex subunit C3
ATP5H	ATP synthase subunit d
ATP5I	ATP synthase subunit e
ATP5J	ATP synthase-coupling factor 6
ATP5J2	ATP synthase subunit f
ATP5K	ATP synthase subunit e
ATP5L	ATP synthase subunit g
ATP5O	ATP synthase subunit O
ATPI	ATP synthase inhibitory factor
ATPIF1	ATP synthase inhibitory factor subunit 1
ATPM	ATP synthase subunit alpha, mitochondrial
ATPMB	ATP synthase subunit beta, mitochondrial
ATPSB	ATP synthase subunit beta
BHAK	Bcl-2 homologous antagonist killer
BAK	BCL2 antagonist killer 1
BAP	B-cell receptor-associated protein
BAP31	B cell receptor associated protein 31
BAX	BCL2 associated X, apoptosis regulator
BCAP31	B cell receptor associated protein 31
BCL2	B-cell lymphoma 2, apoptosis regulator
BCL2A1	BCL2 related protein A1
BCL2L11	BCL2 like protein 11
BCL2L3	BCL2 like protein 3
BCL2L4	BCL2 like protein 4
BCL2L5	BCL2 like protein 5
BCL2L7	BCL2 like protein 7
BCR	Breakpoint cluster region protein
BFL1	Bcl-2-related gene expressed in fetal liver
BID	BH3 interacting domain death agonist
BIK	BCL2 interacting killer
BIM	Bcl-2 Interacting Mediator of cell death
C11orf83	chromosome 11 open reading frame 83
C14orf151	chromosome 14 open reading frame 151
C14orf173	chromosome 14 open reading frame 73
C15orf13	chromosome 15 open reading frame 13
C19orf70	chromosome 19 open reading frame 70
C1orf151	chromosome 1 open reading frame 151
C1orf166	chromosome 1 open reading frame 166
C1QBP	complement C1q binding protein
C20orf52	chromosome 20 open reading frame 52
C2orf33	chromosome 2 open reading frame 33
C9orf54	chromosome 9 open reading frame 54
CAL1H	Calpactin I heavy polypeptide Protein
LPC2D	Lipocortin II
CAL1L	Calpactin-1 light chain
CALR	calreticulin
CCDC56	Cytochrome c oxidase assembly factor 3 homolog
CCT5	Chaperonin Containing TCP1 Subunit 5
CCTE	Chaperonin Containing TCP1, Subunit epsilon
CDM	Caldesmon
CDN1	synonym for Bcl-2 homologous antagonist/killer
CGI-106	comparative gene identification 106
CGI-135	comparative gene identification 135
CGI-51	comparative gene identification 51
CGI-61	comparative gene identification 61
CHCHD3	coiled-coil-helix-coiled-coil-helix domain containing 3
CLP11	human gene encoding p11
CMAR	Cell matrix adhesion regulator
COA3	cytochrome c oxidase assembly factor 3
COX4	Cytochrome c oxidase subunit 4 isoform 1
COX4I1	cytochrome c oxidase subunit 4I1
COX4I2	Cytochrome c oxidase subunit 4 isoform 2
COX5A	Cytochrome c oxidase subunit 5A
COX5B	Cytochrome c oxidase subunit 5B
COX6A1	Cytochrome c oxidase subunit 6A1
COX6A2	Cytochrome c oxidase subunit 6A2
COX6B1	Cytochrome c oxidase subunit 6B1
COX6B2	Cytochrome c oxidase subunit 6B2
COX6C	Cytochrome c oxidase subunit 6C
COX7A1	Cytochrome c oxidase subunit 7A1
COX7A2	Cytochrome c oxidase subunit 7A2
COX7B	Cytochrome c oxidase subunit 7B
COX7B2	Cytochrome c oxidase subunit 7B2
COX7C	Cytochrome c oxidase subunit 7C
COX8A	Cytochrome c oxidase subunit 8A
COX8C	cytochrome c oxidase subunit 8C
CPRP1	synonym of Mitofusin 2
CRTC	Calreticulin
CXorf33	Chromosome X open reading frame 33
CYC1	Ubiquinol-Cytochrome-C Reductase Complex Cytochrome C1 Subunit
CYPA	Peptidyl-prolyl cis-trans isomerase A
DA	dopaminergic
DAPIT	Diabetes-associated protein in insulin-sensitive tissues
DDAH	dimethylarginine dimethylaminohydrolase
DDAH1	dimethylarginine dimethylaminohydrolase 1
DDOST	dolichyl-diphosphooligosaccharide--protein glycosyltransferase non-catalytic subunit
DHC1	Cytoplasmic dynein 1 heavy chain 1
DIC	Mitochondrial dicarboxylate carrier
DJ-1	Protein/nucleic acid deglycase DJ-1
DLP1	dynamin like protein 1
DNA	Deoxyribonucleic acid
DNAJC19	DnaJ heat shock protein family (Hsp40) member C19
DNCH1	Cytoplasmic dynein 1 heavy chain 1
DNCL	Dynein light chain 1
DNECL	Cytoplasmic dynein 1 heavy chain 1
DNM1L	dynamin 1 like
DNM2	dynamin 2
DRP1	dynamin related protein 1
DXS1357E	p28 synonym
DYHC	Cytoplasmic dynein 1 heavy chain 1
DYN2	dynamin 2
DYNC1H1	dynein cytoplasmic 1 heavy chain 1
EC	Enzyme Commission number
EEF1A	eukaryotic translation elongation factor 1 alpha 1
EEF1A1	eukaryotic translation elongation factor 1 alpha 1
EEF1B	Elongation factor 1-beta
EEF1B2	Elongation factor 1-beta
EF1A	Elongation factor 1-alpha 1
EF1B	Elongation factor 1-beta
EFE2	endomyocardial fibroelastosis
EIF5A	eukaryotic translation initiation factor 5A
ER	endoplasmic reticulum
ERM	ezrin/moesin/radixin
ERP57	Endoplasmic reticulum resident protein 57
ERP60	Endoplasmic reticulum resident protein 60
FAM121A	Family With Sequence Similarity 121A
FAM121B	Family With Sequence Similarity 121A
FAM73A	Family With Sequence Similarity 73A
FAM73B	Family With Sequence Similarity 73B
FIS1	fission, mitochondrial 1
FKBP4	FK506 binding protein 4
FKBP52	Peptidyl-prolyl cis-trans isomerase FKBP4
FLN2	filamin 2
FLNC	filamin C
FLOT1	flotillin 1
FTSH1	ATP-dependent zinc metalloprotease YME1L1
FUBP1	far upstream element binding protein 1
FUNDC1	FUN14 domain containing 1
G4.5	synonym of Tafazzin
GARS	glycyl-tRNA synthetase
GC1QBP	complement C1q binding protein
GC2 B415	Glutamate carrier 2
GDAP1	ganglioside induced differentiation associated protein 1
GIDE	Mitochondrial ubiquitin ligase activator of NFKB 1
GIG20	growth-inhibiting gene 20
GIG42	growth-inhibiting gene 42
GL004	synonym of MFF
GRP58	58 kDa glucose-regulated protein
GRP75	75 kDa glucose-regulated protein
GRP78	78 kDa glucose-regulated protein
GRS	Glasgow rearranged sequence
GSTK1	glutathione S-transferase kappa 1
GSTO1	glutathione S-transferase omega 1
GSTTLP28	Glutathione S-transferase omega-1
HABP1	Hyaluronan Binding Protein 1
HACBP	High Affinity Calcium-Binding Protein
HBEBP2	HBEAG-binding protein 2
HBPA1	hematopoietic BCL2-related protein A1
HC8	human proteasome alpha-subunit C8
HCVFTP2	HCV F-Transactivated Protein 2
HDCMD47P	synonym of Glutathione S-transferase Subunit 13
hfzo1	human fuzzy onions 1
HIPPIE	Human Integrated Protein-Protein Interaction rEference web tool
HMP	synonym for inner membrane mitochondrial protein
HSC70	Heat shock cognate 71 kDa protein
HSP27	Heat shock protein beta-1
HSP28	Heat shock protein beta-1
HSP60	heat shock protein family D (Hsp60)
HSP73	Heat shock cognate 71 kDa protein
HSP90AB1	heat shock protein 90 alpha family class B member 1
HSP90B	heat shock protein 90 alpha family class B
HSPA10	Heat shock cognate 71 kDa protein
HSPA1L	heat shock protein family A (Hsp70) member 1 like
HSPA5	heat shock protein family A (Hsp70) member 5
HSPA8	heat shock protein family A (Hsp70) member 8
HSPA9	heat shock protein family A (Hsp70) member 9
HSPA9B	heat shock protein family A (Hsp70) member 9
HSPB1	heat shock protein family B (small) member 1
HSPC009	ORF name for Cytochrome c oxidase assembly factor 3 homolog
HSPC108	ORF name for Stomatin-like protein 2
HSPC2	Heat shock protein HSP 90-beta
HSPC242	ORF name for Mitochondrial fission process protein 1
HSPC263	ORF name for Ubiquitin thioesterase OTUB1
HSPCB	Heat shock protein HSP 90-beta
HSPD1	heat shock protein family D (Hsp60) member 1
IF(1)	Inhibitor Of F(1)F(O)-ATPase
ILVBL	gene ilvB acetolactate synthase like
IMM	inner mitochondrial membrane
IMMT	inner membrane mitochondrial protein
IMS	intermembrane space
INF2	protein inverted formin 2
IPS1	Interferon beta promoter stimulator protein 1
KIAA0098	synonym of CCT5
KIAA0115	synonym of DDOST
KIAA0214	synonym of Mitofusin-2
KIAA0313	synonym of RAPGEF2
KIAA0325	synonym of DYNC1H1
KIAA0348	synonym of SYNJ2
KIAA0491	synonym of SH3GLB1
KIAA0549	synonym of TRAK2
KIAA0567	synonym of Dynamin-like 120 kDa protein
KIAA1042	synonym of TRAK1
KIAA1271	synonym of MAVS
KIAA1848	synonym of SH3GLB2
LDHB	lactate dehydrogenase B
LEM6	Ligand effect modulator 6
LENG7	Elongation factor 1-alpha 1
LETM1	leucine zipper and EF-hand containing transmembrane protein 1
LGALS1	Lectin galactoside-binding soluble 1
LMN1	Prelamin-A/C
LMNA	Lamin A
MADH2	Mothers against decapentaplegic homolog 2
MADR2	Mad-related protein 2 Protein
MAP3K5	Mitogen-activated protein kinase kinase kinase 5
MAPL	Mitochondrial ubiquitin ligase activator of NFKB 1
MARCH5	membrane associated ring-CH-type finger 5
MAVS	mitochondrial antiviral signaling protein
MCL1	Induced myeloid leukemia cell differentiation protein Mcl-1
MDH2	malate dehydrogenase 2
MFF	mitochondrial fission factor
MFN1	mitofusin 1
MFN2	mitofusin 2
MIC 27	synonym for MICOS complex subunit MIC27
MIC10	synonym for MICOS complex subunit MI10
MIC13	synonym for MICOS complex subunit MIC13
MIC19	synonym for MICOS complex subunit MIC19
MIC23	synonym for MICOS complex subunit MIC23
MIC26	synonym for MICOS complex subunit MIC26
MIC60	synonym for MICOS complex subunit MIC60
MICOS	mitochondrial contact site and cristae organizing system
MID49	Mitochondrial dynamics protein MID49
MID51	Mitochondrial dynamics protein MID51
MIEF1	mitochondrial elongation factor 1
MIEF2	mitochondrial elongation factor 2
MIG10	Abnormal cell migration protein 10
MIG5	Abnormal cell migration protein 5
MIGA1	mitoguardin 1
MIGA2	mitoguardin 2
MINOS1	mitochondrial inner membrane organizing system 1
MINOS2	Mitochondrial Inner Membrane Organizing System 2
MINOS3	Mitochondrial Inner Membrane Organizing System 3
Mito_shape	Mitochondrial shaping proteins
MITRAC12	Cytochrome c oxidase assembly factor 3 homolog
MPP+	1-methyl-4-phenylpyridinium
MPRP1	multidrug resistance protein 1
MPTP	1-methyl-4-phenyl-1,2,3,6-tetrahydropyridine
MT-ATP6	Mitochondrially Encoded ATP Synthase 6
MT-ATP8	Mitochondrially Encoded ATP Synthase 8
MT-CO1	Mitochondrially Encoded Cytochrome C Oxidase I
MT-CO2	Mitochondrially Encoded Cytochrome C Oxidase II
MT-CO3	Mitochondrially Encoded Cytochrome C Oxidase III
MT-CYB	Mitochondrially Encoded Cytochrome B
MTFP1	mitochondrial fission process 1
mt-HSP70	mitochondrial heat shock protein family A (Hsp70)
MTP18	Mitochondrial fission process protein 1
MUL1	mitochondrial E3 ubiquitin protein ligase 1
MULAN	Mitochondrial ubiquitin ligase activator of NFKB 1
My022	ORF name for Mitochondrial fission process protein 1
My025	ORF name for MICOS complex subunit MIC26
My032	ORF name for ATP synthase subunit d
MYL6	myosin light chain 6
NAD	nicotinamide adenine dinucleotide oxidized
NADH	nicotinamide adenine dinucleotide reduced
NBK	Bcl-2-interacting killer
NDUFA10	NADH:ubiquinone oxidoreductase subunit A10
NDUFA11	NADH:ubiquinone oxidoreductase subunit A11
NDUFA4	NADH Dehydrogenase (Ubiquinone) 1 Alpha Subcomplex, 4
NDUFS1	NADH:ubiquinone oxidoreductase core subunit S1
NDUFS3	NADH:ubiquinone oxidoreductase core subunit S3
NEDD8	neural precursor cell expressed, developmentally down-regulated 8
NFE2L2	nuclear factor, erythroid 2 like 2
NFKB	nuclear factor kappa-light-chain-enhancer of activated B cells
NHERF	Na(+)/H(+) exchange regulatory cofactor NHE-RF1
NHERF1	Na(+)/H(+) exchange regulatory cofactor NHE-RF1
NHERF-1 protein	Na(+)/H(+) exchange regulatory cofactor NHE-RF1
NIMA	never in mitosis gene a
NO	nitric oxide
Nox1	NADPH oxidase 1
NPM	nucleophosmin
NPM1	nucleophosmin 1
NRAPGEP	Rap guanine nucleotide exchange factor 2
NRF1	nuclear respiratory factor 1
NRF2	Nuclear factor erythroid 2-related factor 2
OAT	ornithine aminotransferase
OGDH	oxoglutarate dehydrogenase
OIP106	O-linked *N*-acetylglucosamine transferase interacting protein 106
OK/SW-cl.110	ORF name for Phosphoglycerate kinase 1
OK/SW-cl.56	ORF name for Tubulin beta chain
OK/SW-cl.82	ORF name for 40S ribosomal protein S15a
OMA1	Overlapping Activity With M-AAA Protease
OMM	outer mitochondrial membrane
OPA1	Optic Atrophy Protein 1
OST48 OK/SW-cl.45	synomym for Dolichyl-diphosphooligosaccharide--protein glycosyltransferase 48 kDa subunit
OTB1	Ubiquitin thioesterase OTUB1
OTU1	Ubiquitin thioesterase OTUB1
OTUB1	OTU deubiquitinase, ubiquitin aldehyde binding 1
OXPHOS	oxidative phosphorylation
PARK2	E3 ubiquitin-protein ligase parkin
PARL	presenilin associated rhomboid like
PD	Parkinson’s disease
PD04912	ORF name for Up-regulated during skeletal muscle growth protein 5
PDIA3	protein disulfide isomerase family A member 3
PDZGEF1	Rap guanine nucleotide exchange factor 2
PERC	Peroxisome proliferator-activated receptor gamma coactivator 1-beta
PGAM5	Serine/threonine-protein phosphatase PGAM5
PGC1	Peroxisome proliferator-activated receptor gamma coactivator 1-alpha
PGC1A	Peroxisome proliferator-activated receptor gamma coactivator 1-alpha
PGC1B	Peroxisome proliferator-activated receptor gamma coactivator 1-beta
PGK1	phosphoglycerate kinase 1
PGKA	Phosphoglycerate kinase 1
PGN	paraplegin
PHB	prohibitin
PHB2	prohibitin 2
PIG4	ORF name for MICOS complex subunit MIC60
PIG52	ORF name for MICOS complex subunit MIC60
PIN1	peptidylprolyl cis/trans isomerase, NIMA-interacting 1
PINK1	PTEN-induced putative kinase 1
PLD6	phospholipase D family member 6
PP578	ORF name for Endophilin-B2
PPARGC1	Peroxisome proliferator-activated receptor gamma coactivator 1-alpha
PPARGC1A	Peroxisome proliferator-activated receptor gamma coactivator 1-alpha
PPARGC1B	Peroxisome proliferator-activated receptor gamma coactivator 1-beta
PPIA	peptidylprolyl isomerase A
PRDX4	peroxiredoxin 4
PRELI	Protein Of Relevant Evolutionary And Lymphoid Interest
PRELID1	PRELI domain-containing protein 1
PRI	synonym for Ribonuclease inhibitor
PRKN	parkin RBR E3 ubiquitin protein ligase
PRO0903	ORF name for Serum albumin
PRO1708	ORF name for Serum albumin
PRO2044	ORF name for Serum albumin
PRO2207	ORF name for Presenilins-associated rhomboid-like protein
PRO2619	ORF name for Serum albumin
PRO2675	ORF name for Serum albumin
PSARL	Presenilins-associated rhomboid-like protein
PSC8	Proteasome component C8
PSEC0112	ORF name for Mitoguardin 2
PSMA3	proteasome subunit alpha 3
PTPN5	protein tyrosine phosphatase, non-receptor type 5
QIL1	synonym for Chromosome 19 Open Reading Frame 70
RAB14	Ras-related protein Rab-14
RAB2	Ras-related protein Rab2
RAB2A	Ras-related protein Rab-2A
RAC1	Ras-related C3 botulinum toxin substrate 1
RAPGEF2	Rap guanine nucleotide exchange factor 2
REA	synonym for Prohibitin-2
RHO12	ras homolog family member
RHOA	ras homolog family member A
RING-type E3	really interesting new gene type E3
RNF153	Ring Finger Protein 153
RNF218	Ring Finger Protein 218
RNH	Ribonuclease inhibitor
RNH1	ribonuclease/angiogenin inhibitor 1
ROMO1	reactive oxygen species modulator 1
ROS	reactive oxygen species
RPN1	Ribophorin I
RPN2	ribophorin II
RPS15A	ribosomal protein S15a
RPS3, OK/SW-cl.26	symbol and ORF name for 40S ribosomal protein S3
S100A10	S100 calcium binding protein A10
SAM50	Sorting and assembly machinery component 50 homolog
SAMM50	Sorting and assembly machinery component 50 homolog
SBBI12	ORF name for PRELI domain-containing protein 1
SBP	selenium binding protein
SELENBP1	selenium binding protein 1
SF2P32	nuclear splicing factor
SFXN1	sideroflexin 1
SH3	SRC Homology 3 Domain
SH3GLB1	SH3 domain containing GRB2 like, endophilin B1
SH3GLB2	SH3 domain containing GRB2 like, endophilin B2
SIR2L	SIR2-like protein
SIR2L2	SIR2-like protein 2
SIRT2	sirtuin 2
SLC20A4	solute carrier family 20 member 4
Slc25A	solute carrier family 25
SLC25A10	solute carrier family 25 member 10
SLC25A11	solute carrier family 25 member 11
SLC25A12	solute carrier family 25 member 12
SLC25A13	solute carrier family 25 member 13
SLC25A18	solute carrier family 25 member 18
SLC25A38	solute carrier family 25 member 38
SLC9A3R1	solute carrier family 9 (sodium/hydrogen exchanger), member 3 regulator 1
SLP2	Stomatin-like protein 2
SMAD2	Mothers against decapentaplegic homolog 2
SMCR7	Smith-Magenis Syndrome Chromosomal Region Candidate Gene 7 Protein
SMCR7L	Smith-Magenis Syndrome Chromosomal Region Candidate Gene 7 Protein-like
SN	Substantia nigra
SPG7	Paraplegin
SSBP	single stranded DNA binding protein
SSBP1	single stranded DNA binding protein 1
STOML2	stomatin like 2
SYNJ2	synaptojanin 2
TAZ	tafazzin
TC25	Ras-Like Protein TC25
TCA	tricarboxylic acid cycle
TCF6	Transcription factor
TCF6L2	Transcription Factor 6-Like 2
TCP-1-epsilon	T-complex protein 1 subunit epsilon
TFAM	transcription factor A, mitochondrial
TIM14	Mitochondrial Import Inner Membrane Translocase Subunit TIM14
TIMM14	Translocase Of Inner Mitochondrial Membrane 14
TMSA	Tropomyosin alpha-1 chain
TMSB	tropomyosin Beta
TPD52	tumor protein D52
TPM1	tropomyosin 1
TPM2	tropomyosin 2
TPM3	tropomyosin 3
TPM4	tropomyosin 4
TRAK1	trafficking kinesin protein 1
TRAK2	trafficking kinesin protein 2
TRG3	transformation-related gene 3
TTC11	tetratricopeptide Repeat Domain 11
TUBA1A	tubulin alpha 1a
TUBA3	tubulin alpha-3 chain
TUBB	tubulin beta class I
TUBB2C	Tubulin beta-4B chain
TUBB4B	tubulin beta 4B class IVb
TUBB5	Tubulin beta chain
TUFM	Tu translation elongation factor, mitochondrial
UniProt	Universal Protein Resource
UNQ1866/PRO4302	ORF name for MICOS complex subunit MIC26
UNQ1868/PRO4304	ORF name for ATP-dependent zinc metalloprotease YME1L1
UNQ655/PRO1286	ORF name for Ubiquinol-cytochrome-c reductase complex assembly factor 3
UNQ696/PRO1341	ORF name for Serum albumin
UNQ8193/PRO23204	ORF for MICOS complex subunit MIC27
UQBP	Ubiquinol-cytochrome c reductase complex 14 kDa protein
UQCC3	ubiquinol-cytochrome c reductase complex assembly factor 3
UQCR10	Ubiquinol-Cytochrome C Reductase, Complex III Subunit X
UQCR11	Ubiquinol-Cytochrome C Reductase, Complex III Subunit XI
UQCRB	ubiquinol-cytochrome c reductase binding protein
UQCRC1	ubiquinol-cytochrome c reductase core protein 1
UQCRC2	ubiquinol-cytochrome c reductase core protein 2
UQCRFS1	ubiquinol-cytochrome c reductase, Rieske iron-sulfur polypeptide 1
UQCRFS1	ubiquinol-cytochrome c reductase, Rieske iron-sulfur polypeptide 1
UQCRFS1P1	ubiquinol-cytochrome c reductase, Rieske iron-sulfur polypeptide 1 pseudogene 1
UQCRFSL1	Ubiquinol-cytochrome c reductase Rieske iron-sulfur subunit pseudogene 1
UQCRH	ubiquinol-cytochrome c reductase hinge protein
UQCRQ	ubiquinol-cytochrome c reductase complex ubiquinone-binding protein QP-C
USMG5	Up-regulated during skeletal muscle growth protein 5
VAT1	vesicle amine transport 1
VCP	valosin containing protein
VDAC1	Voltage-dependent anion-selective channel protein 1
VDAC2	voltage dependent anion channel 2
VDAC3	voltage dependent anion channel 3
VDAC	Voltage-dependent anion-selective channel
VIM	vimentin
VISA	Virus-induced-signaling adapter
YME1L	YME1-like protein 1
YME1L1	YME1-like protein 1
YWHAE	tyrosine 3-monooxygenase/tryptophan 5-monooxygenase activation protein epsilon
YWHAZ	tyrosine 3-monooxygenase/tryptophan 5-monooxygenase activation protein zeta
